# Facile and innovative catalytic protocol for intramolecular Friedel–Crafts cyclization of Morita–Baylis–Hillman adducts: Synergistic combination of chiral (salen)chromium(III)/BF_3_·OEt_2_ catalysis

**DOI:** 10.3762/bjoc.17.140

**Published:** 2021-08-26

**Authors:** Karthikeyan Soundararajan, Helen Ratna Monica Jeyarajan, Raju Subimol Kamarajapurathu, Karthik Krishna Kumar Ayyanoth

**Affiliations:** 1 Organic and Material Chemistry Research Laboratory, The American College, Madurai, Tamil Nadu, India; 2 Department of Chemistry, Fatima College, Madurai, Tamil Nadu, India

**Keywords:** boron trifluoride etherate, chiral (salen)chromium(III), intramolecular Friedel–Crafts cyclization, Morita–Baylis–Hillman adducts, substituted-1*H*-indenes

## Abstract

The chiral (salen)Cr(III)/BF_3_·OEt_2_ catalytic combination was found to be an effective catalyst for intramolecular Friedel–Crafts cyclization of electron-deficient Morita–Baylis–Hillman adducts. In presence of mild reaction conditions the chiral (salen)Cr(III)/BF_3_·OEt_2_ complex affords 2-substituted-1*H*-indenes from unique substrates of Morita–Baylis–Hillman adducts via an easy operating practical procedure.

## Introduction

Reactions associated with carbon–carbon bond formations are explored for their synthetic utility in extending the carbon framework in organic molecules [1–3]. Among the known C–C bond forming methodologies the Friedel–Crafts reaction is the most utilized methodology. As a result of its broad scope of applications in inter/intraorganic molecular transformations, researchers are interested in creating novel, mild and efficient Friedel–Crafts methodologies. In spite of its worthwhile synthetic applications the Friedel–Crafts (FC) reaction faces a shortcoming and major challenge in operating with electron-deficient arenes [4–7]. Operating FC reactions in versatile synthons such as Morita–Baylis–Hillman (MBH) adducts is a challenging process. Inter/intramolecular Friedel–Crafts reactions of Morita–Baylis–Hillman adducts leads to a variety of products such as quinolinones [8], cycloheptene-6-carboxylates [9], indenes [10–13] and indanones [14]. However, most of the reported Friedel–Crafts reactions utilize either strong Lewis acid catalysts or severe reaction conditions resulting in low yield, unwanted byproducts and tedious workup methodologies [15–16]. Therefore, developing an efficient, novel and efficient Friedel–Crafts reaction methodology in presence of novel catalysts will widely serve its purpose in synthesising molecules of biological interest. Thereby with the goal of operating FC reactions in terminal allylic units of Morita–Baylis–Hillman adducts we sought to screen the M(salen) complexes.

**Scheme 1:**
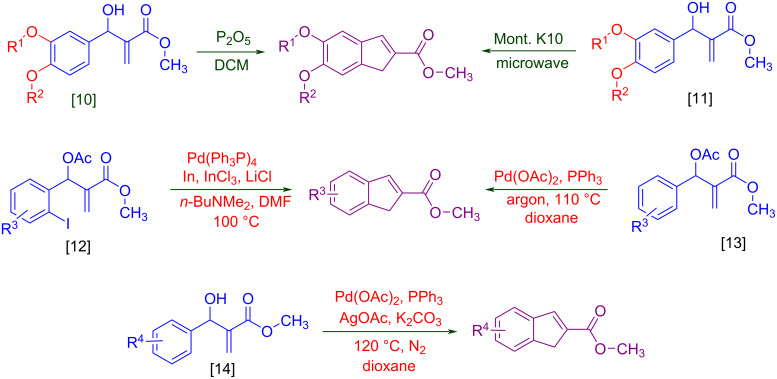
Literature-reported approaches to synthesise indene from MBH adducts [10–14].

Intramolecular Friedel–Crafts cyclizations in Morita–Baylis–Hillman adducts are known to undergo annulations generating cyclic frameworks of indene. To the best of our knowledge, five reports are cited for the intramolecular FC reaction in MBH adducts. The first report was by Basavaiah et al. [10] on the phosphorous pentoxide-catalysed synthesis of indene using alkoxy-substituted MBH adducts. Later Shanmugam et al. [11] reported a Mont. K10-catalysed synthesis of indenes from similar alkoxy MBH adducts. Thereafter Lee et al. [12] and Xu et al. [13] synthesised indenes in presence of a palladium catalyst from allylic acetates of MBH adducts. Recently Anas et al. [14] has reported the palladium-catalysed synthesis of indene from MBH adducts at 120 °C under N_2_ atmosphere. The methodologies referred in Scheme 1 access indenes either relying on harsh reaction conditions or on the dialkoxy and *ortho*-halide- substituted MBH adducts. Thereby alternative synthetic routes should be accessed to overcome the harsh reaction conditions, structural requirements and scalability issues intrinsically associated with the literature reported procedures. Hence chiral catalysts could possibly be utilized for the current investigation with expectations to minimize the formation of regioisomers, dimerized side products and overcome the usage of allylic-OH protected MBH adducts.

Metallosalen complexes can be easily prepared and safely handled. The stability of metallosalen complexes convinced us to explore them as suitable chiral Lewis acid catalysts for the Friedel–Crafts cyclization of MBH adducts. Mononuclear(salen) complexes of aluminium, chromium, manganese and cobalt were chosen and screened for the current investigation.

## Results and Discussion

To evaluate the scope of the intramolecular Friedel–Crafts cyclization protocol the Morita–Baylis–Hillman adduct **5a**, was surveyed as the model substrate. The optimization experiments are listed in Table 1. The cyclization of MBH adduct **5a** was examined in presence of metal(III)–salen complex (5 mol %) as catalysts and BF_3_·OEt_2_ (2.5 mol %) as co-catalyst. Though all metal–salen complexes catalysed the reaction (Table 1, entries 1–4), but the [Cr(III)salenCl]/BF_3_·OEt_2_ combination promoted the cyclization effectively (45%, Table 1, entry 4). Regardless of Lewis acid character, BF_3_·OEt_2_ provides a number of undesired byproducts in absence of [Cr(III)salenCl] complex (Table 1, entry 5). In absence of co-catalyst or additives Cr(III)salen complexes are known to effectively promote cyclization reactions [17–18]. In contrast to the literature reports the Cr(III)–salen complex in absence of co-catalyst BF_3_·OEt_2_ provides the expected product at a very low yield of 21% (Table 1, entry 6). Therefore, the consequence of increasing [Cr(salen)Cl] catalyst loading from 10 mol % to 20 mol % (Table 1, entries 7–9) in presence of co-catalyst BF_3_·OEt_2_ was examined. Convincingly the analysis revealed that an optimum loading of 15 mol % of [Cr(salen)Cl] catalyst was sufficient to access indene from MBH adducts (Table 1, entry 8). In order to analyse the role of the BF_3_·OEt_2_ in promoting the synthesis of indenes, two optimization reactions at a co-catalyst loading of 1.5 mol and 3 mol % were attempted (Table 1, entries 10 and 11). Though these reactions promoted the transformation of Morita–Baylis–Hillman adducts to the corresponding indenes, however, their yields were comparatively lower. It is thereby evident that a co-catalyst load of 2.5 mol % is crucial for the synthesis of indene. However, unexpectedly Cr(salen) complexes with non-coordinating counter ions BF_4_ and AsF_6_ delivers indene only at a moderate yield of 61% and 56%, respectively (Table 1, entries 12 and 13). We further investigated the role of co-catalysts (2.5 mol %), viz., AlCl_3_, SnCl_2_, FeCl_3_ and ZnCl_2_ with [Cr(salen)Cl] (15 mol %) in promoting the Friedel–Crafts cyclization in MBH adduct **5a** (Table 1, entries 14–17). Interestingly among all co-catalysts examined, boron trifluoride etherate was the found to be the suitable co-catalyst in accelerating the proposed reaction. Furthermore on screening the optimized catalytic combination in presence of alternative solvents (Table 1, entries 18–22) recommended dichloromethane as the most preferable solvent (81%, Table 1, entry 16).

**Table 1:** Optimization of reaction condition for intramolecular Friedel–Crafts cyclization in Morita–Baylis–Hillman adducts.

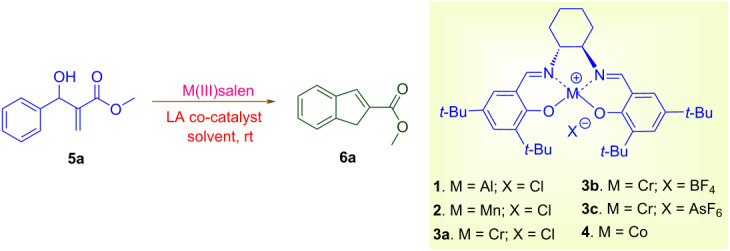					

Entry	M(salen)	Co-catalyst	Time (h)	Solvent	Yield (%)

1	Al(III)salen (5 mol %)	BF_3_·OEt_2_ (2.5 mol %)	12	CHCl_3_	18
2	Mn(III)salen (5 mol %)	BF_3_·OEt_2_ (2.5 mol %)	12	CHCl_3_	25
3	Co(II)salen (5 mol %)	BF_3_·OEt_2_ (2.5 mol %)	12	CHCl_3_	36
4	Cr(III)salen (5 mol %)	BF_3_·OEt_2_ (2.5 mol %)	12	CHCl_3_	45
5	–	BF_3_·OEt_2_ (2.5 mol %)	24	CHCl_3_	10
6	Cr(III)salen (5 mol %)	–	24	CHCl_3_	21
7	Cr(III)salen (10 mol %)	BF_3_·OEt_2_ (2.5 mol %)	12	CHCl_3_	53
8	Cr(III)salen (15 mol %)	BF_3_·OEt_2_ (2.5 mol %)	12	CHCl_3_	72
9	Cr(III)salen (20 mol %)	BF_3_·OEt_2_ (2.5 mol %)	12	CHCl_3_	73
10	Cr(III)salen (15 mol %)	BF_3_·OEt_2_ (1.5 mol %)	12	CHCl_3_	36
11	Cr(III)salen (15 mol %)	BF_3_·OEt_2_ (3 mol %)	12	CHCl_3_	65
12	Cr(III)salen (15 mol %)	BF_3_·OEt_2_ (2.5 mol %)	12	CHCl_3_	61^a^
13	Cr(III)salen (15 mol %)	BF_3_·OEt_2_ (2.5 mol %)	12	CHCl_3_	56^b^
14	Cr(III)salen (15 mol %)	AlCl_3_ (2.5 mol %)	12	CHCl_3_	57
15	Cr(III)salen (15 mol %)	SnCl_2_ (2.5 mol %)	12	CHCl_3_	36
16	Cr(III)salen (15 mol %)	FeCl_3_ (2.5 mol %)	12	CHCl_3_	22
17	Cr(III)salen (15 mol %)	ZnCl_2_ (2.5 mol %)	12	CHCl_3_	18
**18**	**Cr(III)salen (15 mol %)**	**BF** **_3_** **·OEt** **_2 _** **(2.5 mol %)**	**12**	**CH** **_2_** **Cl** **_2_**	**81**
19	Cr(III)salen (15 mol %)	BF_3_·OEt_2_ (2.5 mol %)	12	THF	49
20	Cr(III)salen (15 mol %)	BF_3_·OEt_2_ (2.5 mol %)	12	MeCN	57
21	Cr(III)salen (15 mol %)	BF_3_·OEt_2_ (2.5 mol %)	12	toluene	32
22	Cr(III)salen (15 mol %)	BF_3_·OEt_2_ (2.5 mol %)	12	diethyl ether	12

^a^ – BF_4_, ^b^ – AsF_6_.

By considering the efficiency and ease of the transformation, the optimized reaction conditions were attempted for a series of structurally distinct MBH adducts (1 mmol), at a catalyst load of **3a** (15 mol %)/BF_3_·OEt_2_ (2.5 mol %) in 10 mL dichloromethane (Table 2). A series of indene derivatives were obtained from moderate to excellent yield (58–85%). The steric hindrance and electronic effect of electron-withdrawing substituents such as nitriles and methyl/ethyl esters did not significantly affect the scope of the reaction. On contradictory to the literature reports [Cr(III)salenCl] complex surprisingly catalysed the intramolecular Friedel–Crafts reaction in electron deactivated arenes of MBH adducts [10–14]. However, *ortho*/*meta*-substituted arenes of MBH adducts were inert to the Cr(salen)/BF_3_·OEt_2_ catalytic system (**5m**–**r**, Table 2). This could be an outcome of steric hindrance exerted by the substitutents at the arene moiety of the MBH adducts.

**Table 2:** Scope of Morita–Baylis–Hillman adducts.^a^

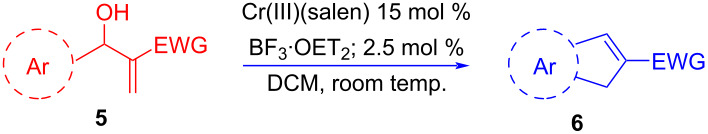			

Reactant	Product (yield)	Reactant	Product (yield)

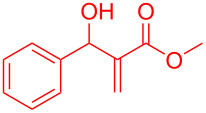 **5a**	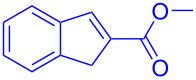 **6a** (81%)	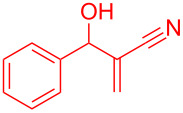 **5b**	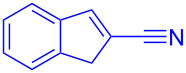 **6b** (58%)
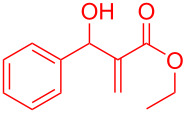 **5c**	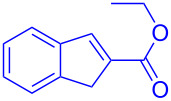 **6c** (67%)	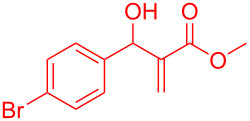 **5d**	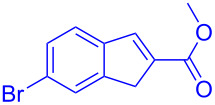 **6d** (74%)
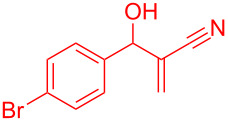 **5e**	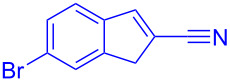 **6e** (60%)	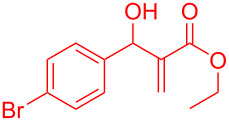 **5f**	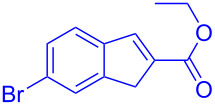 **6f** (68%)
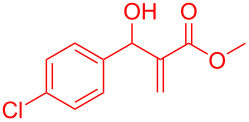 **5g**	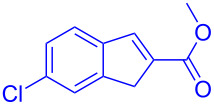 **6g** (80%)	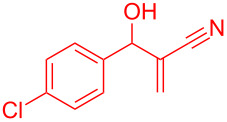 **5h**	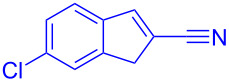 **6h** (65%)
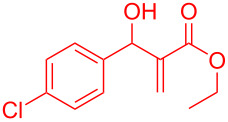 **5i**	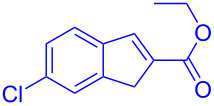 **6i** (72%)	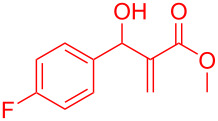 **5j**	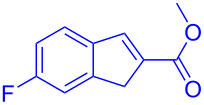 **6j** (85%)
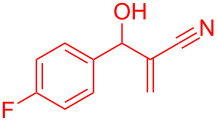 **5k**	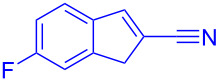 **6k** (69%)	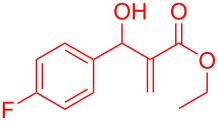 **5l**	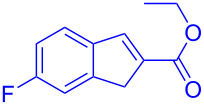 **6l** (76%)
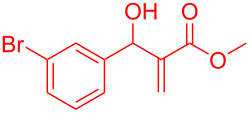 **5m**	–^b^	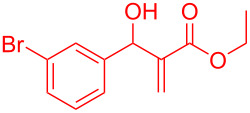 **5n**	–^b^
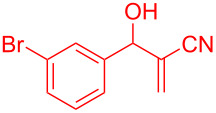 **5o**	–^b^	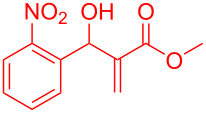 **5p**	–^b^
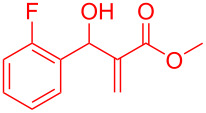 **5q**	–^b^	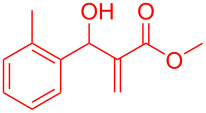 **5r**	–^b^

^a^All reactions shown were performed at 1 mmol scale. Isolated yield of indene, **6** are shown within brackets. ^b^Not obtained.

Thereafter, to inspect the utility of the reaction for a gram-scale reaction, we endeavoured a model reaction using the optimized standard reaction conditions. Convincingly MBH adduct **5h** (10 mmol) delivered the desired product **6h** in 73% yield (1.28 g). The outcome of this practical scale synthesis demonstrates the synthetic utility of the stabilized reaction for large scale synthesis. The structure of the synthesised indene compounds (**6a–f**) were deduced from ^1^H NMR, ^13^C NMR, elemental analysis and high-resolution mass spectrometry.

**Scheme 2:**
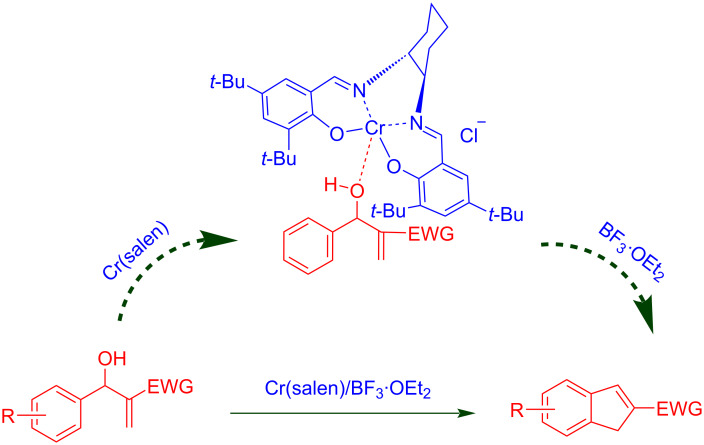
Proposed model for the intramolecular Friedel–Crafts cyclization.

To account for the observed synthesis of indene from MBH adducts a model was suggested based on the reports of Rawal et al. [17,19], Katsuki et al. [20–21] and Jurczak et al. [22]. The proposed model is an outcome of interaction between the Mortia–Baylis–Hillman adduct and the Cr(salen) complex (Scheme 2), The hydroxyl lone pair of the MBH adduct is expected to coordinate with the Cr(salen) complex in a position of minimized steric strain to that of the cyclohexyl group. We anticipate that this arrangement would facilitate the intramolecular Friedel–Crafts cyclization of the MBH adducts.

**Scheme 3:**
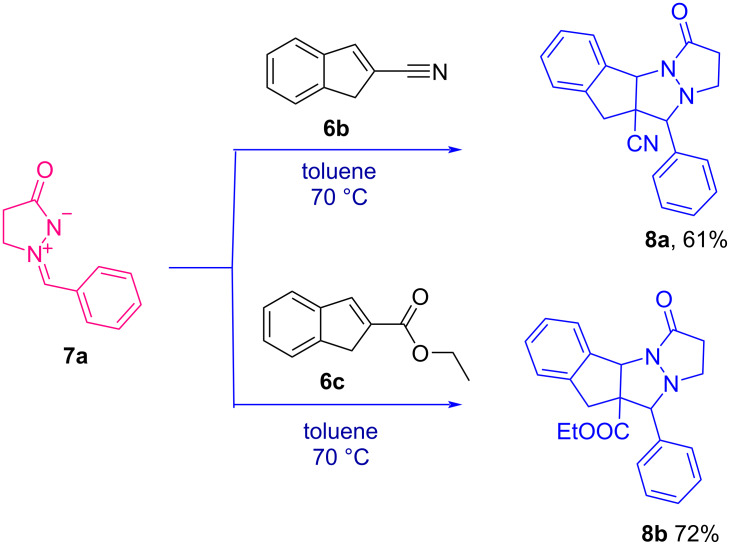
Reactions of azomethine imine **7a** with 2-substituted 1*H*-indenes **6a** and **6b**.

Cycloaddition of azomethine imine **7a** with 2-substituted-1*H*-indenes **6b** and **6c** was attempted to ascertain the structure of the synthesized 1*H*-indenes (Scheme 3). The azomethine imine, 1-benzylidene-3-oxopyrazolidin-1-ium-2-ide (**7a**) was synthesised at room temperature by treating methyl acrylate, hydrazine hydrate and benzaldehyde in a yield of 67%. On treating the synthesised azomethine imine **7a** (1.2 mM) and 2-substituted-1*H*-indenes **6b** and **6c** (1 mM) in toluene at 70 °C affords **8a** and **8b** via [3 + 2] cycloaddition reaction at a yield of 61% and 72%, respectively. Encouraged by the outcome of this non-catalytic cycloaddition reaction, similar studies using 2-substituted-1*H*-indenes (**6a**–**f**) with azomethine imines are underway.

## Conclusion

In summary we have developed an efficient synthetic protocol for intramolecular Friedel–Crafts cyclization in electron-deficient Morita–Baylis–Hillman adducts. The present methodology is attractive because it directly utilizes MBH adducts over the possible reports on utilizing allylic-OH protected MBH adducts [12–13] to access indenes. The chiral Cr(III)(salen)/BF_3_·OEt_2_-catalyzed synthesis of indenes from MBH adducts restricts the formation of regioisomers [10] and hinders the formation of dimerised side products [11]. In addition, the reaction smoothly accesses substituted indenes from MBH adducts at room temperature. In conclusion, the methodology is attractive for its easy transformation of multifaceted MBH adducts into 2-substituted indenes. We have also envisaged that the octahedral complex derived from Cr(salen) and MBH adduct to facilitate intramolecular Friedel–Crafts cyclization.

## Experimental

### Typical procedure for the synthesis of indenes **6a–l**

The Morita–Baylis–Hillman adduct **5a** (1 mmol) and chromium(salen)(III) complex **3a** (15 mol %) were dissolved in 10 mL of dichloromethane in a flame dried 50 mL RB flask. To the reaction mixture BF_3_·OEt_2_ (2.5 mol %) was added drop-wise over 10–15 min using a well-dried glass syringe. The reaction mixture was stirred for 12 hours at room temperature, followed by thin-layer chromatography. The resulting solution was quenched with sodium bicarbonate (20 mL) and the aqueous layer was extracted with dichloromethane (3 × 20 mL). The combined extracts were washed with brine and the organic layer was dried (anhydrous MgSO_4_). After filtration, the solvent was removed under reduced pressure and the crude product was purified on silica gel (using hexane/EtOAc) to afford the desired product **6a** as a white solid (81%).

**Methyl 1*H*-indene-2-carboxylate (6a):** Yield: 160 mg (81%); white solid; mp 85–87 °C; IR (cm^−1^): 3062, 2953, 2884, 1947, 1735, 1640, 1548, 1470, 1442, 1215, 734; ^1^H NMR (CDCl_3_, 300 MHz) δ_H_ 7.99 (s, 1H, alkene-CH), 7.64–7.41 (m, 4H, Aro-H), 4.41 (s, 2H, -CH_2_), 3.87 (s, 3H, COOCH_3_); ^13^C NMR (CDCl_3_, 75 MHz) δ_C_ 168.03, 145.31, 134.67, 130.11, 129.53, 128.62, 128.25, 64.78, 52.24; HRMS (*m*/*z*): [M + H]^+^ calcd for C_11_H_10_O_2_, 174.0681; found, 174.0706

### Synthesis of 1-benzylidene-3-oxopyrazolidin-1-ium-2-ide (**7a**)

A methyl acrylate (0.0225 mol) solution was added drop-wise to the solution of hydrazine monohydrate (0.0205 mol) in 20 mL of ethanol. This solution was stirred at 78 °C for 4 hours; the solvent was evaporated and the residue was dissolved in 5 mL of MeOH. To the residue benzaldehyde (0.0307 mmol) was added and stirred for 14 hours. The solvent was evaporated and the residue was directly purified by column chromatography to afford 1-benzylidenene-3-oxopyrazolidin-1-ium-2-ide as white solid (2.4 g, 67%) [23–24]. Yield: 132 mg (67%); off-white solid; mp 194–196 °C; IR (cm^−1^): 1674, 1652, 1582, 1568, 1454, 1278, 1117; ^1^H NMR (CDCl_3,_ 400 MHz) δ_H_ 8.31–7.46 (m, 5H, Aro-H), 7.14 (s, 1H, N=CH), 4.56–4.50 (t, *J* = 8 Hz, 2H, CO-CH_2_), 2.84–2.79 (t, *J* = 8 Hz, 2H, N-CH_2_); ^13^C NMR (CDCl_3,_ 100 MHz) δ_C_ 185.12 (1C, C=O), 133.02–128.83 (6C, aro-C), 57.92 (1C, N-CH_2_), 29.38 (1C, CO-CH_2_); HRMS (*m*/*z*): [M + H]^+^ calcd for C_10_H_10_N_2_O, 174.0793; found, 174.0818.

### Typical procedure for synthesis of compounds **8a** and **8b**

Indene **6b** (1 mmol) in 2 mL of toluene was successively added to 1-benzylidene-3-oxopyrazolidin-1-ium-2-ide (1.2 mmol). The reaction mixture was stirred at 70 °C for 6 h until the dipolarophile was consumed. After completion of the reaction, toluene was evaporated and extracted using CHCl_3_ and water. The organic layer was dried over anhydrous magnesium sulfate and concentrated in vacuo. Finally, the crude reaction mixture was purified by column chromatography to afford the corresponding [3 + 2] cycloaddition product **8a** in 61% yield.

**Compound (8a):** Yield: 192 mg (61%); yellowish oil; IR (cm^−1^): 2972, 2254, 1954, 1562, 1671, 1455, 1245; ^1^H NMR (CDCl_3_, 400 MHz) δ_H_ 7.80–7.06 (m, 9H, Aro-*H*), 5.90 (s, 1H, *H*C-N-CO), 4.36–4.21 (m, 2H, Aro-C*H_2_*), 3.99–3.95 (m, 1H, Aro-C*H*-N-N), 3.44–3.30 (m, 2H, N-C*H_2_*-CH_2_), 3.05–2.82 (m, 2H, N-CH_2_-C*H_2_*); ^13^C NMR (CDCl_3_, 100 MHz) δ_C_ 165.36, 146.49, 136.10, 133.70, 131.16, 129.66, 129.22, 129.05, 128.94, 128.87, 128.68, 128.21, 127.61, 117.92, 71.69, 68.63, 58.86, 50.22, 45.64, 36.75; HRMS (*m*/*z*): [M + H]^+^ calcd for C_20_H_17_N_3_O, 315.1372; found, 315.1397.

## Supporting Information

File 1 Full experimental details, ^1^H and ^13^C NMR spectra.
