# Supplementary material for: Facile and innovative catalytic protocol for intramolecular Friedel–Crafts cyclization of Morita–Baylis–Hillman adducts: Synergistic combination of chiral (salen)chromium(III)/BF3·OEt2 catalysis
Source: Beilstein J Org Chem. 2021 Aug 26;17:2186–93. doi: 10.3762/bjoc.17.140 (PMC8404216; doi:10.3762/bjoc.17.140)
Supplement: File 1 — Full experimental details, 1H and 13C NMR spectra. [file Beilstein_J_Org_Chem-17-2186-s001.pdf]

BEILSTEIN JOURNAL OF ORGANIC CHEMISTRY

Supporting Information

for

Facile and innovative catalytic protocol for intramolecular Friedel–Crafts cyclization of Morita–Baylis–Hillman adducts: Synergistic combination of chiral (salen)chromium(III)/ $BF_3{\cdot}OEt_2$ catalysis

Karthikeyan Soundararajan, Helen Ratna Monica Jeyarajan,
Raju Subimol Kamarajapurathu and Karthik Krishna Kumar Ayyanoth

Beilstein J. Org. Chem. **2021,** *17,* 2186–2193. doi:10.3762/bjoc.17.140

Full experimental details, ^{1}H and ^{13}C NMR spectra

License and Terms: This is a supporting information file under the terms of the Creative Commons Attribution License (https://creativecommons.org/licenses/by/4.0). Please note that the reuse, redistribution and reproduction in particular requires that the author(s) and source are credited and that individual graphics may be subject to special legal provisions.
The license is subject to the *Beilstein Journal of Organic Chemistry* terms and conditions: (https://www.beilstein-journals.org/bjoc/terms)

Materials and methods

The aldehydes and acrylates were purchased from Sigma-Aldrich Chemicals Private Limited, India and Loba Chemie Private Limited, India. The borontrifluoride etherate, stannous chloride, ferric chloride and zinc chloride were purchased from Sisco Research Laboratories Private Limited, India. Aluminum trichloride was purchased from Loba Chemie Private Limited, India. (*R*,*R*)-N,N'-bis(3,5-di-tert-butylsalicylidene)-1,2 cyclohexanediamino chromium(III) chloride, (*R*,*R*)-(−)-*N,N′*-bis(3,5-di-*tert*-butylsalicylidene)-1,2-cyclohexanediaminomanganese(III) chloride and (*R*,*R*)-*N*,*N′*-bis(3,5-di-*tert*-butylsalicylidene)-1,2-cyclohexanediaminoaluminum(III) chloride were purchased from Sigma-Aldrich Chemicals Private Limited, India. The (*R*,*R*)-*N*,*N′*-bis(3,5-di-*tert*-butylsalicylidene)-1,2-cyclohexanediaminocobalt(II) was purchased from Enantiotech Catalysts Private Limited, India. The catalyst **3b** and **3c** were donated by Orchid Pharma Limited, India. Solvents were purchased from Sisco Research Laboratories Private Limited, India and Merck Life Science Private Limited, India. The high performance thin-layer chromatography plates were purchased from Merck Life Science Private Limited, India. NMR spectra were recorded using a Bruker Avance III HD Nanobay 400 MHz FT-NMR spectrometer at the Gandhigram Rural Institute, India. NMR spectra were recorded using $CDCl_3$ as a solvent. The splitting patterns are designated as s (singlet), d (doublet), q (quartet) and m (multiplet) and coupling constants are expressed in Hz. Chemical shifts for protons and carbons are reported in parts per million (ppm) downfield from tetramethylsilane (TMS) and are internal reference of $CDCl_3$ which resonates at δ 7.26 ppm in ^{1}H NMR and 77.2 ppm in carbon NMR analysis. The infra-red spectra were recorded using JASCO FT/IR-4000 series in the American College, India. High-resolution mass spectra were recorded on Waters XEVO-G2-XS-QToF at Vellore Institute of Technology, India. Melting points of the synthesized compounds were recorded using melting point apparatus of Technico Laboratory products, India.

Proton NMR of methyl 1*H*-indene-2-carboxylate

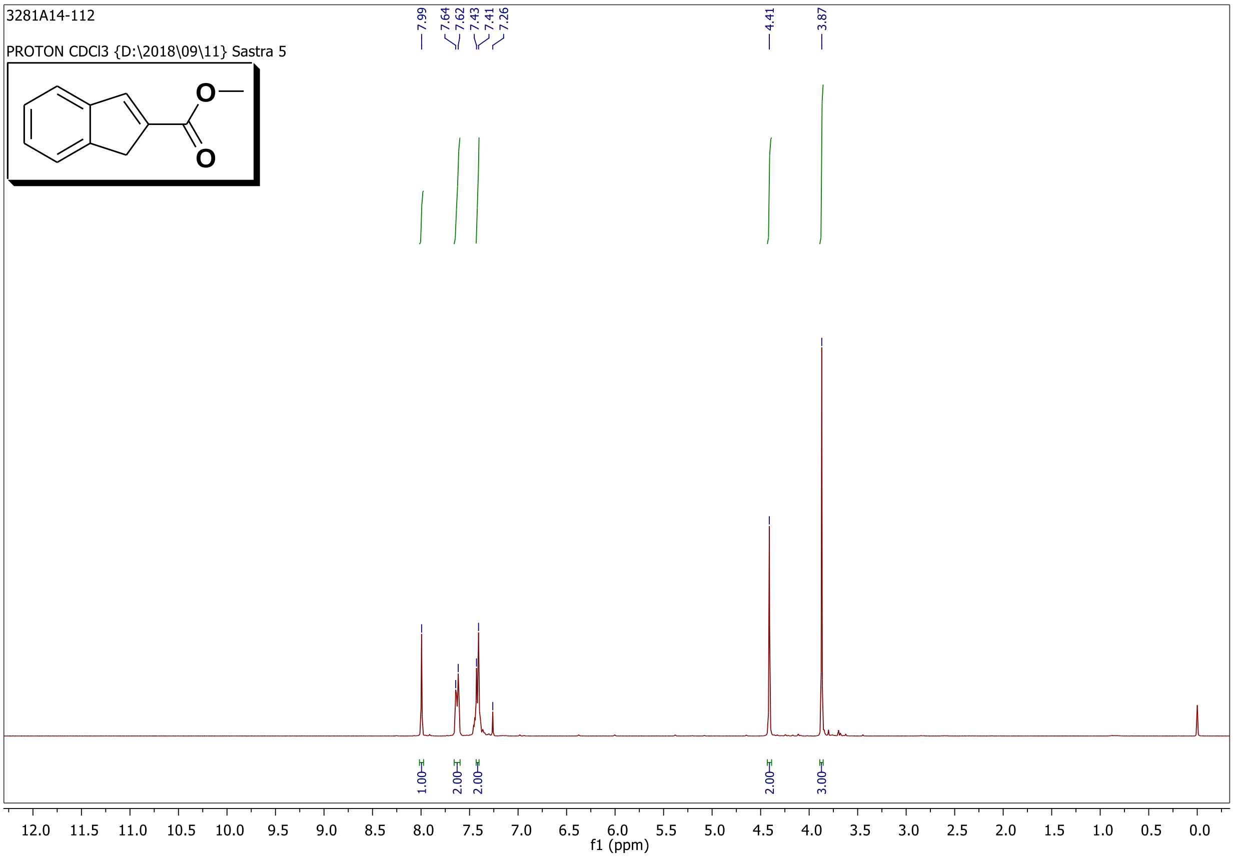

Fig. S1 ^{1}H NMR spectrum of methyl 1*H*-indene-2-carboxylate (**6a**).

Carbon NMR of methyl 1*H*-indene-2-carboxylate

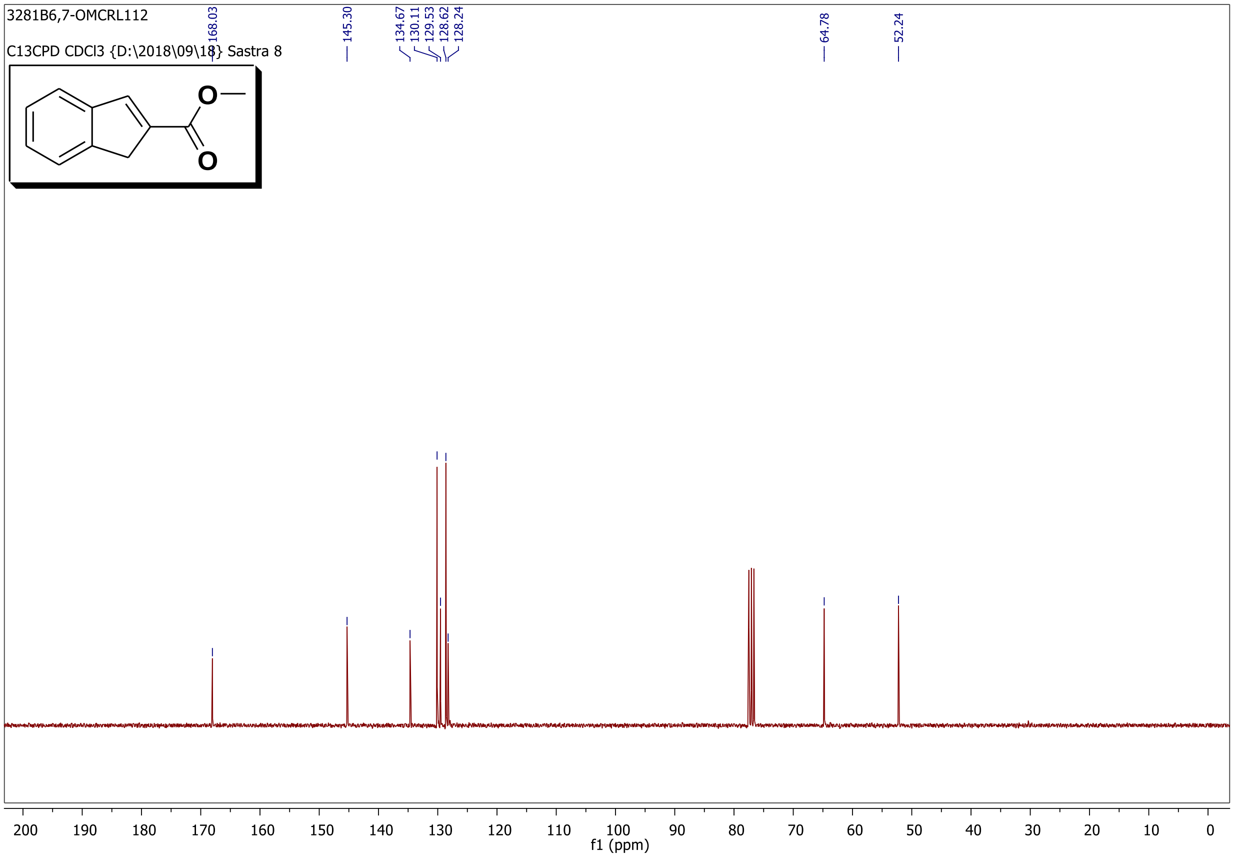

Fig. S2 ^{13}C NMR spectrum of methyl 1*H*-indene-2-carboxylate (**6a**).

Proton NMR of 1*H*-indene-2-carbonitrile

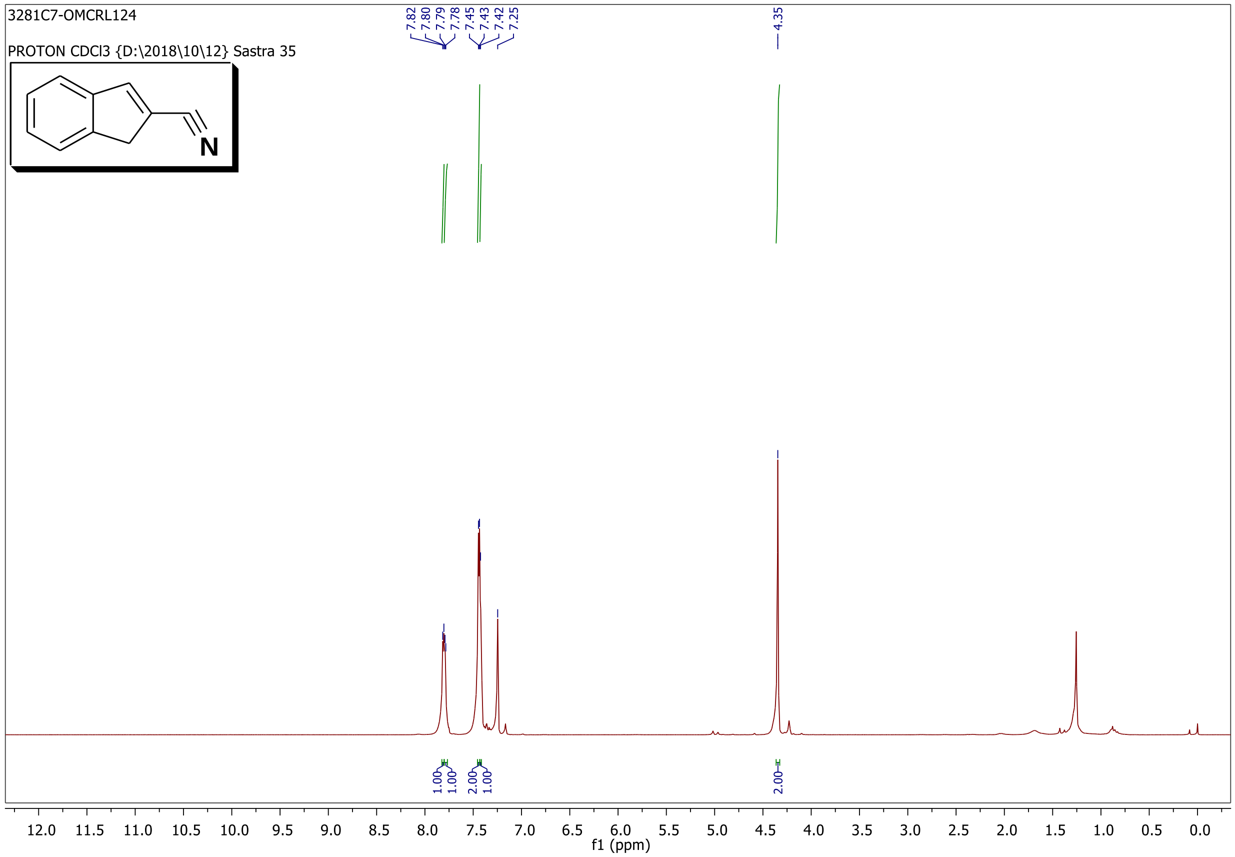

Fig. S3 ^{1}H NMR spectrum 1*H*-indene-2-carbonitrile (**6b**).

Carbon NMR of 1*H*-indene-2-carbonitrile

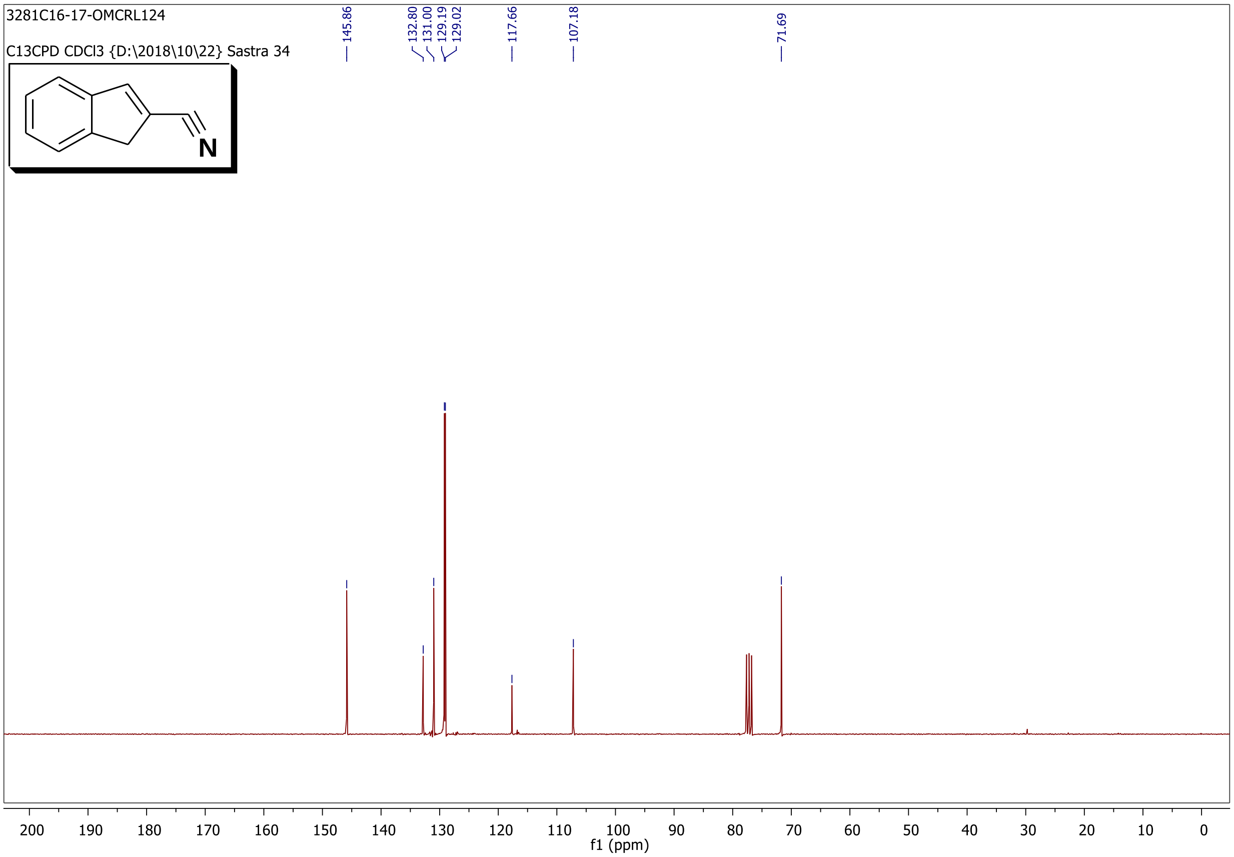

Fig. S4 ^{13}C NMR spectrum 1*H*-indene-2-carbonitrile (**6b**).

Proton NMR of ethyl 1*H*-indene-2-carboxylate

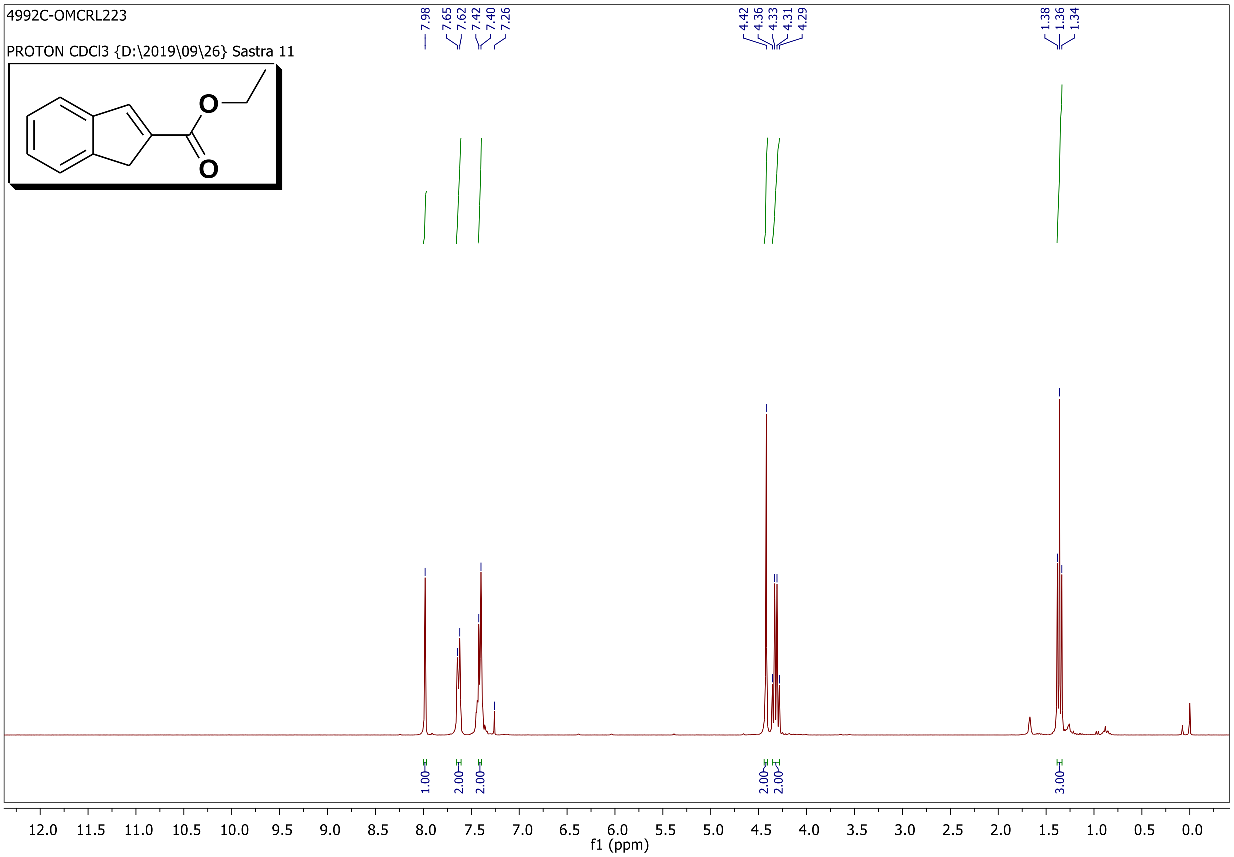

Fig. S5 ^{1}H NMR spectrum of ethyl 1*H*-indene-2-carboxylate (**6c**).

Carbon NMR of ethyl 1*H*-indene-2-carboxylate

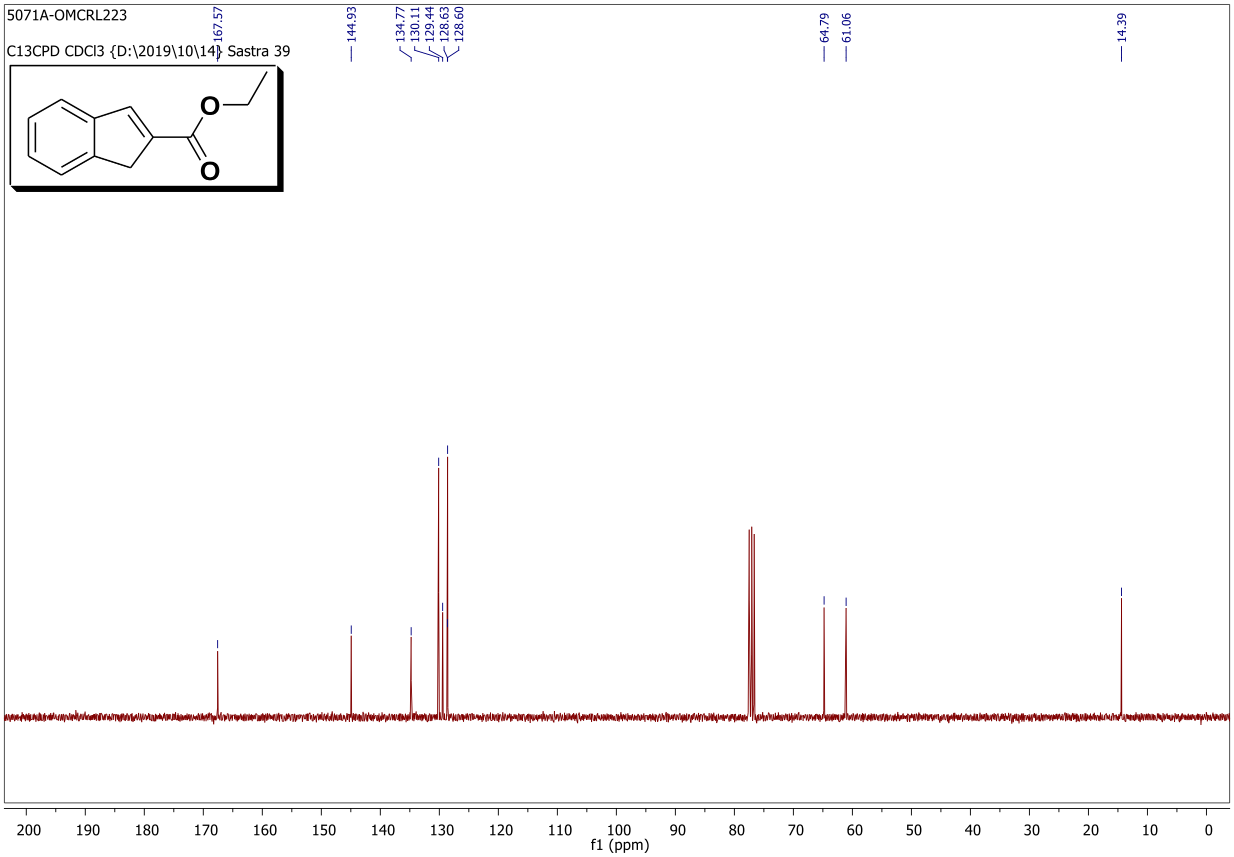

Fig. S6 ^{13}C NMR spectrum of ethyl 1*H*-indene-2-carboxylate (**6c**).

Proton NMR of methyl 6-bromo-1*H*-indene-2-carboxylate

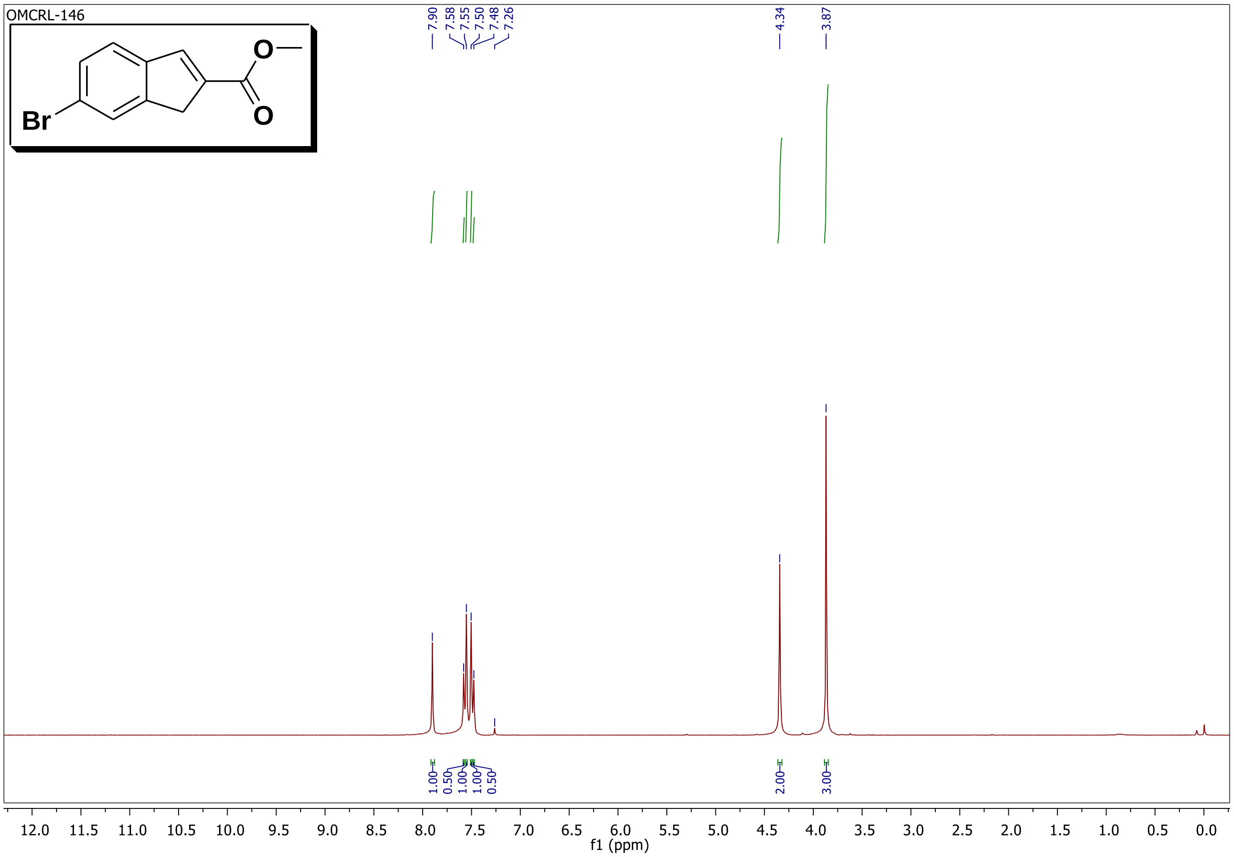

Fig. S7 ^{1}H NMR spectrum of methyl 6-bromo-1*H*-indene-2-carboxylate (**6d**).

Carbon NMR of methyl 6-bromo-1*H*-indene-2-carboxylate

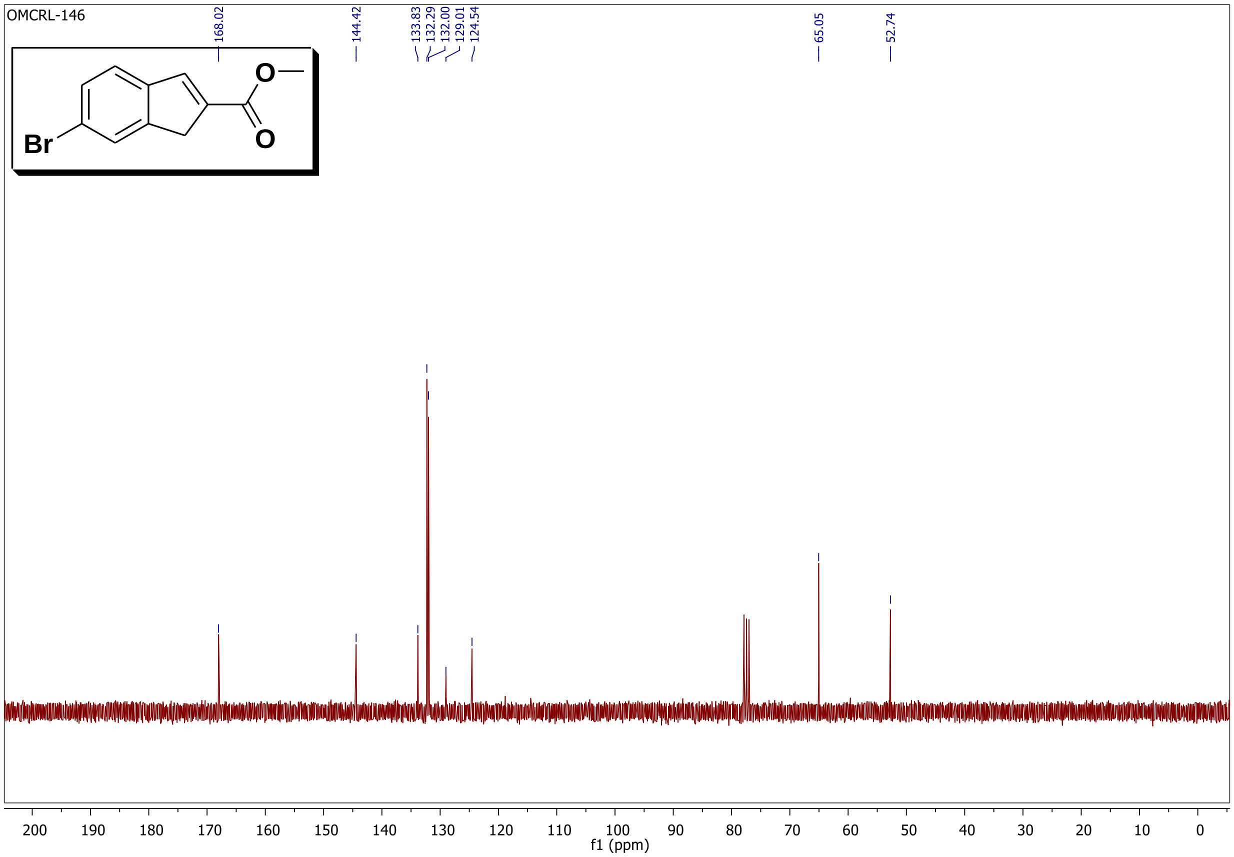

Fig. S8 ^{13}C NMR spectrum of methyl 6-bromo-1*H*-indene-2-carboxylate (**6d**)

Proton NMR of 6-bromo-1*H*-indene-2-carbonitrile

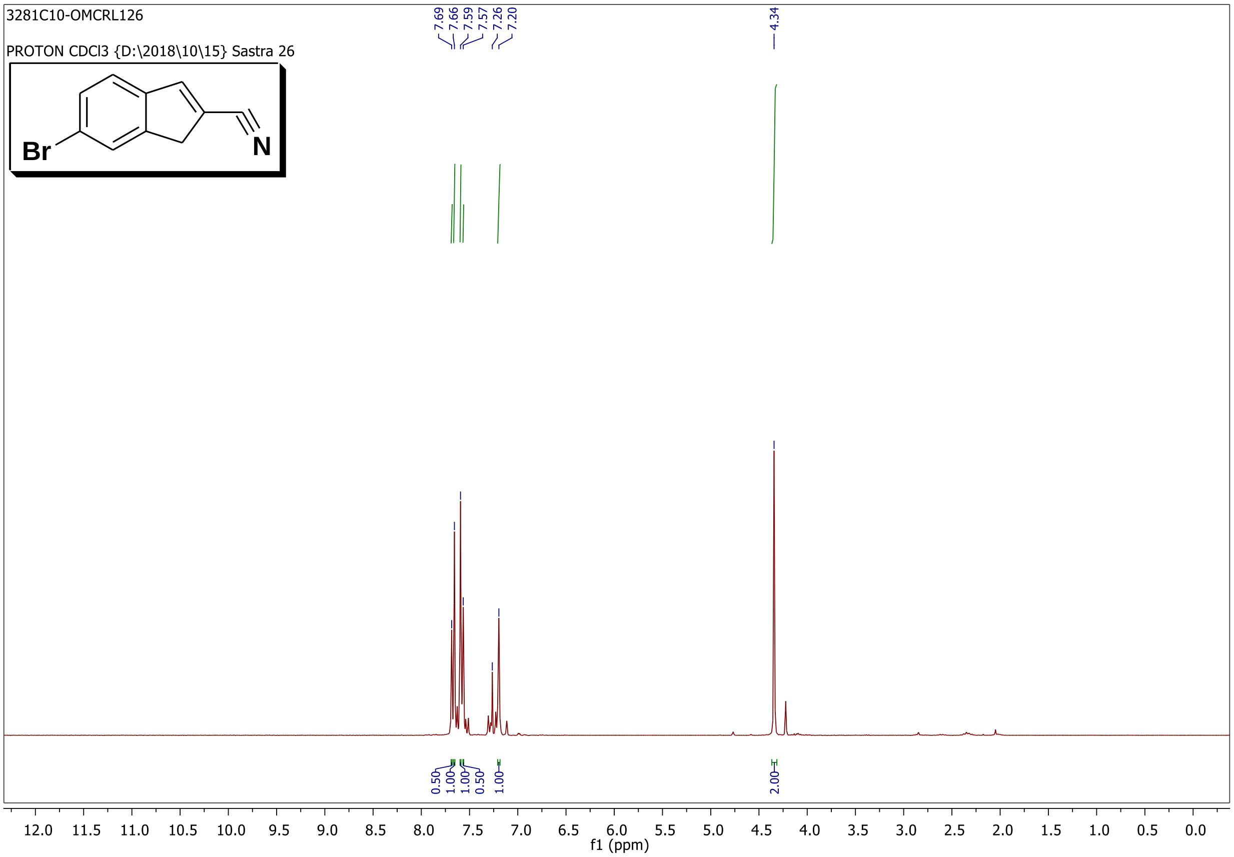

Fig. S9 ^{1}H NMR spectrum 6-bromo-1*H*-indene-2-carbonitrile (**6e**).

Carbon NMR of 6-bromo-1*H*-indene-2-carbonitrile

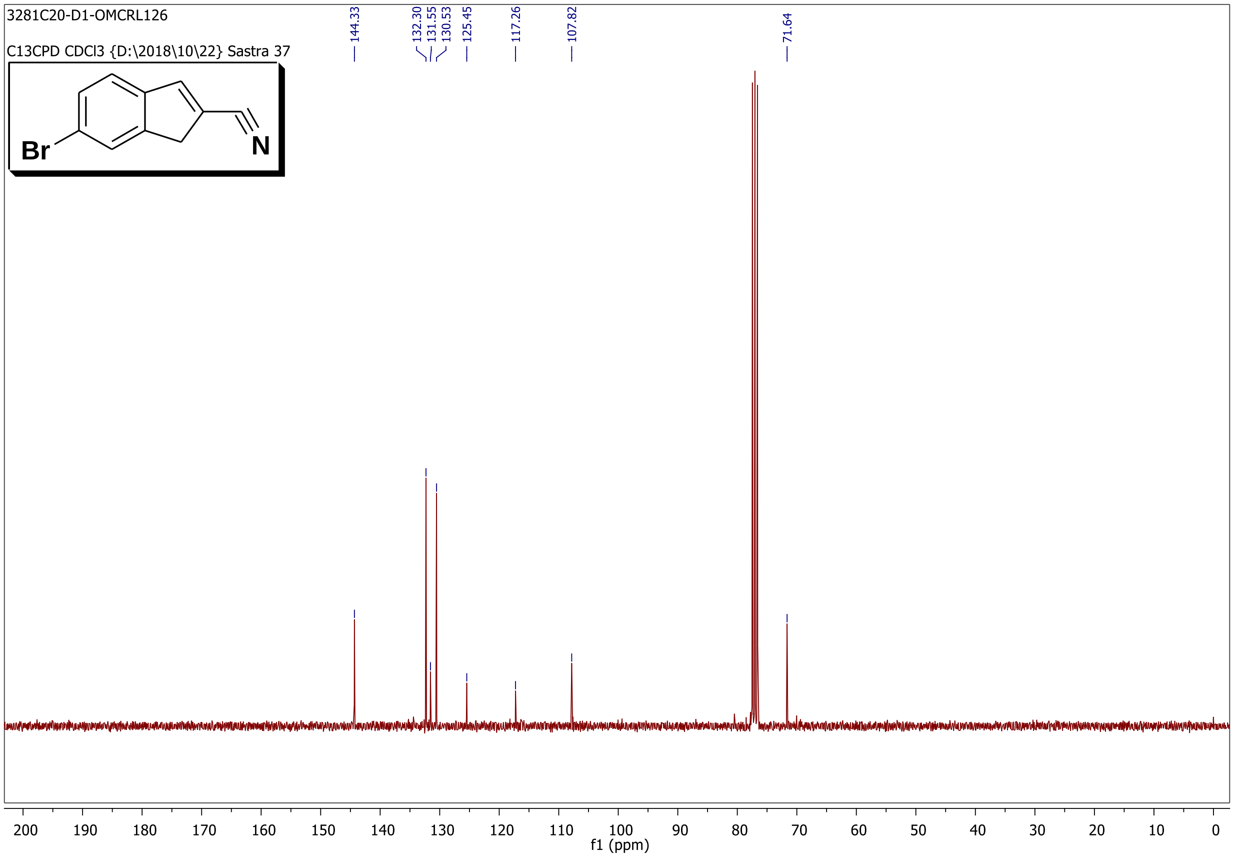

Fig. S10 ^{13}C NMR spectrum 6-bromo-1*H*-indene-2-carbonitrile (**6e**).

<u>**Proton NMR of ethyl 6-bromo-1*H*-indene-2-carboxylate**</u>

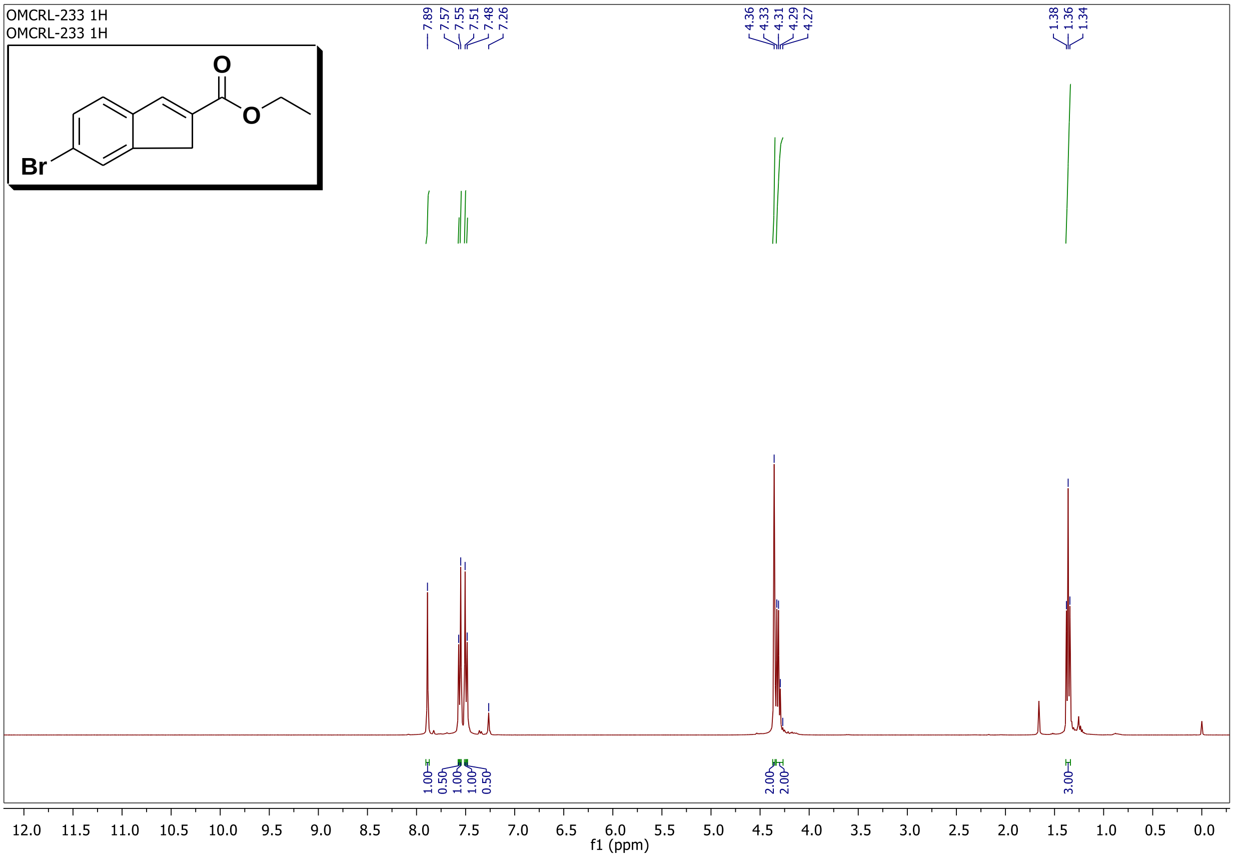

Fig. S11 ^{1}H NMR spectrum of ethyl 6-bromo-1*H*-indene-2-carboxylate (**6f**).

Carbon NMR of ethyl 6-bromo-1*H*-indene-2-carboxylate

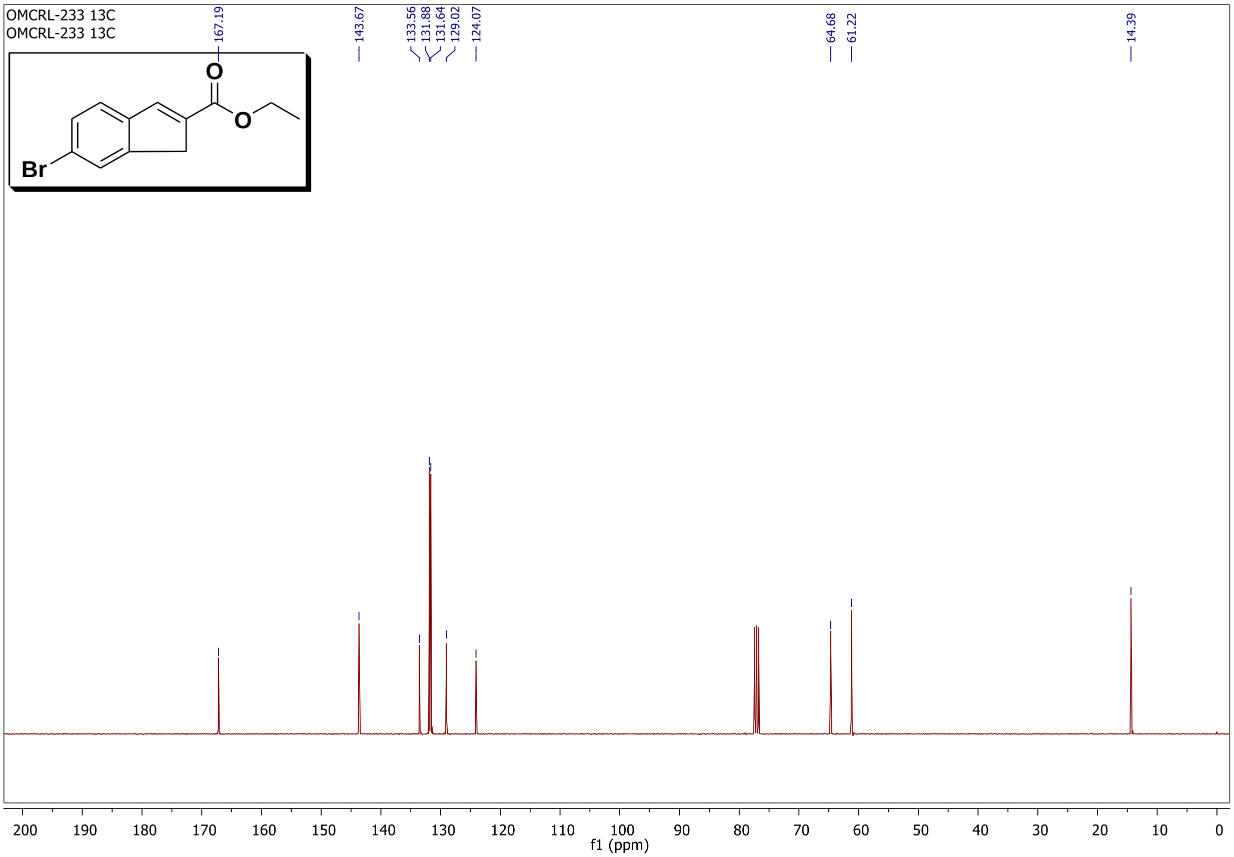

Fig. S12 ^{13}C NMR spectrum of ethyl 6-bromo-1*H*-indene-2-carboxylate (**6f**).

Proton NMR of methyl 6-chloro-1*H*-indene-2-carboxylate

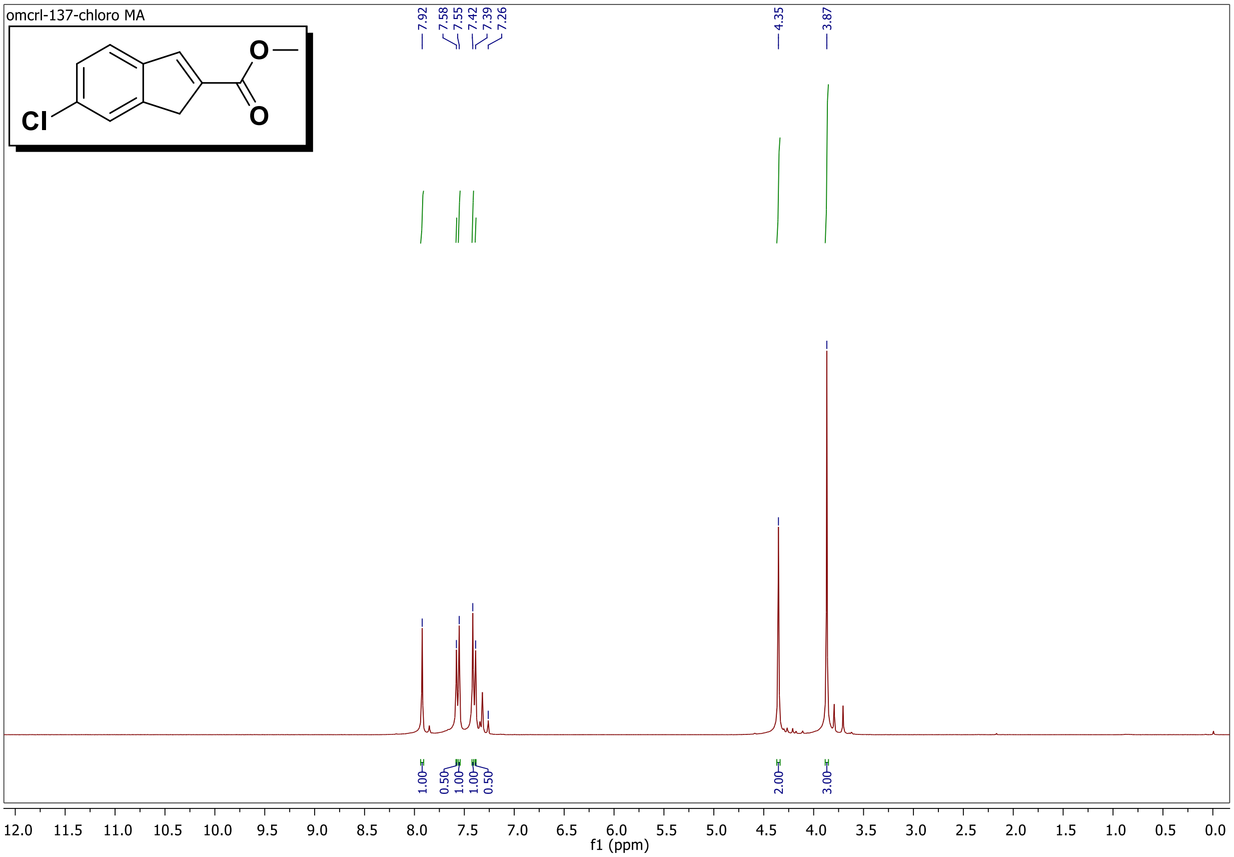

Fig. S13 ^{1}H NMR spectrum of methyl 6-chloro-1*H*-indene-2-carboxylate (**6g**).

Carbon NMR of methyl 6-chloro-1*H*-indene-2-carboxylate

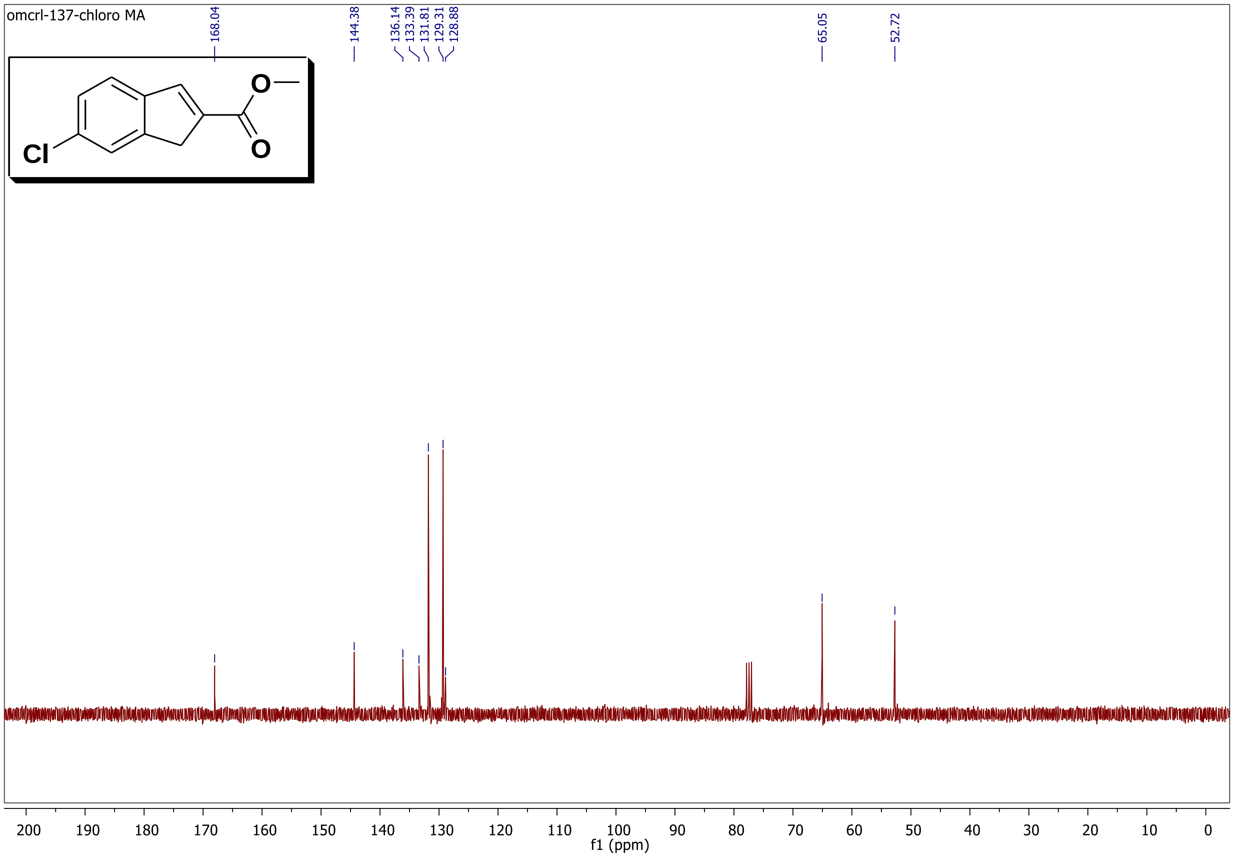

Fig. S14 ^{13}C NMR spectrum of methyl 6-chloro-1*H*-indene-2-carboxylate (**6g**).

Proton NMR of 6-chloro-1*H*-indene-2-carbonitrile

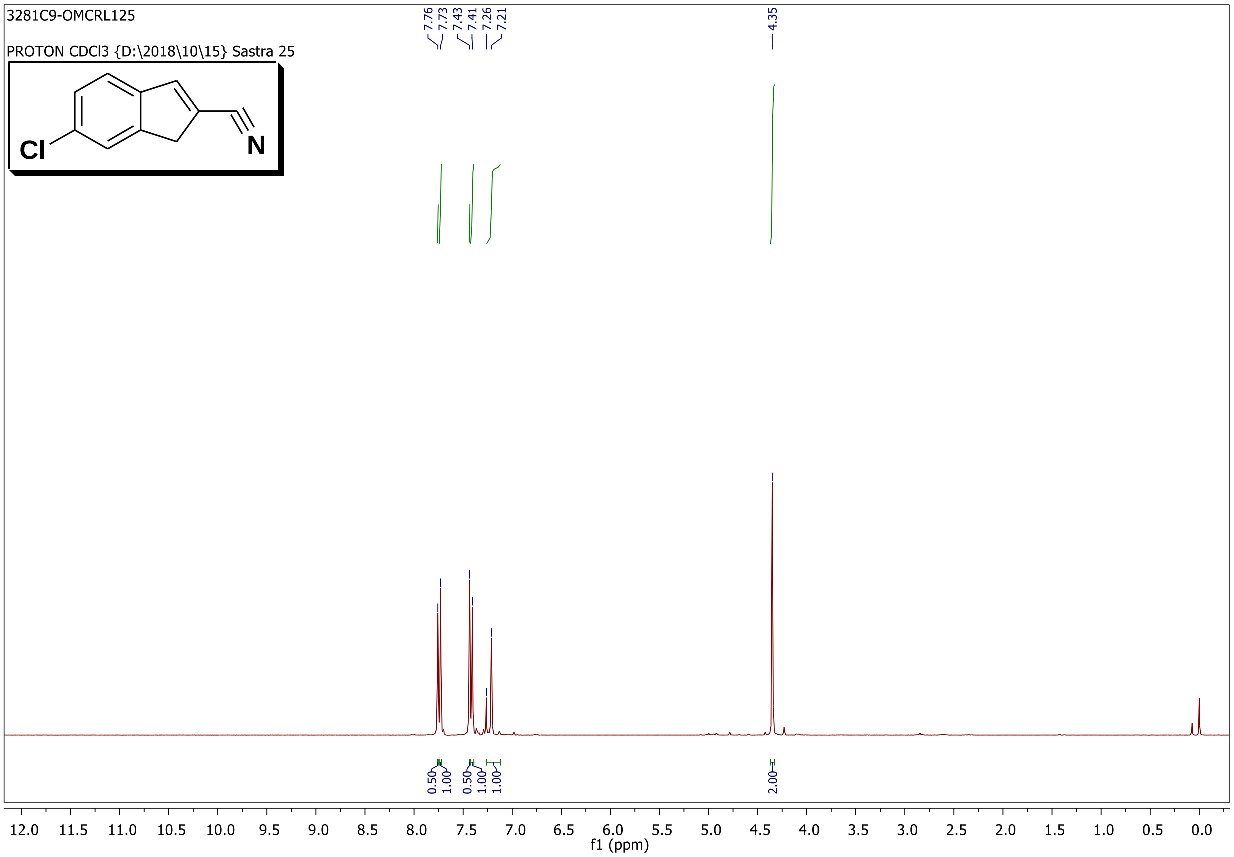

Fig. S15 ^{1}H NMR spectrum 6-chloro-1*H*-indene-2-carbonitrile (**6h**).

Carbon NMR of 6-chloro-1*H*-indene-2-carbonitrile

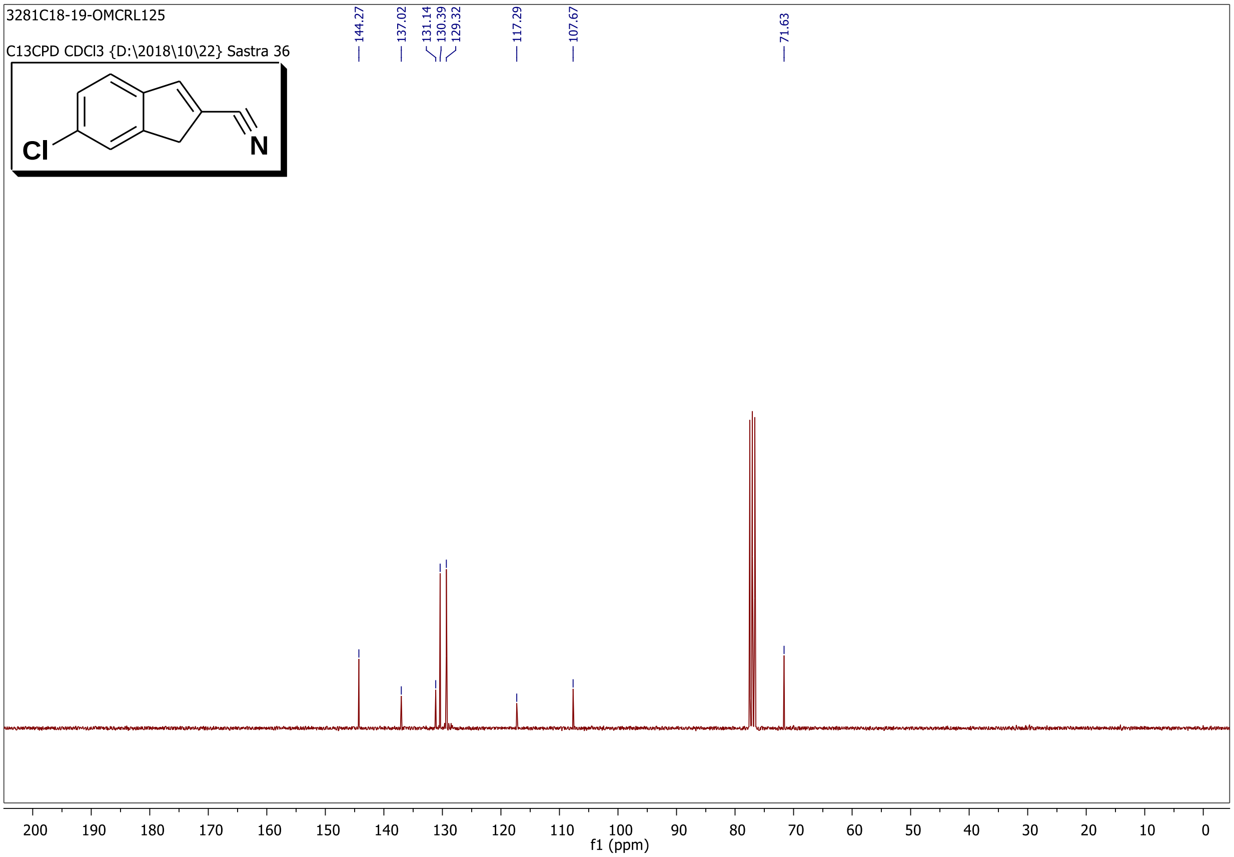

Fig. S16 ^{13}C NMR spectrum 6-chloro-1*H*-indene-2-carbonitrile (**6h**).

Proton NMR of ethyl 6-chloro-1*H*-indene-2-carboxylate

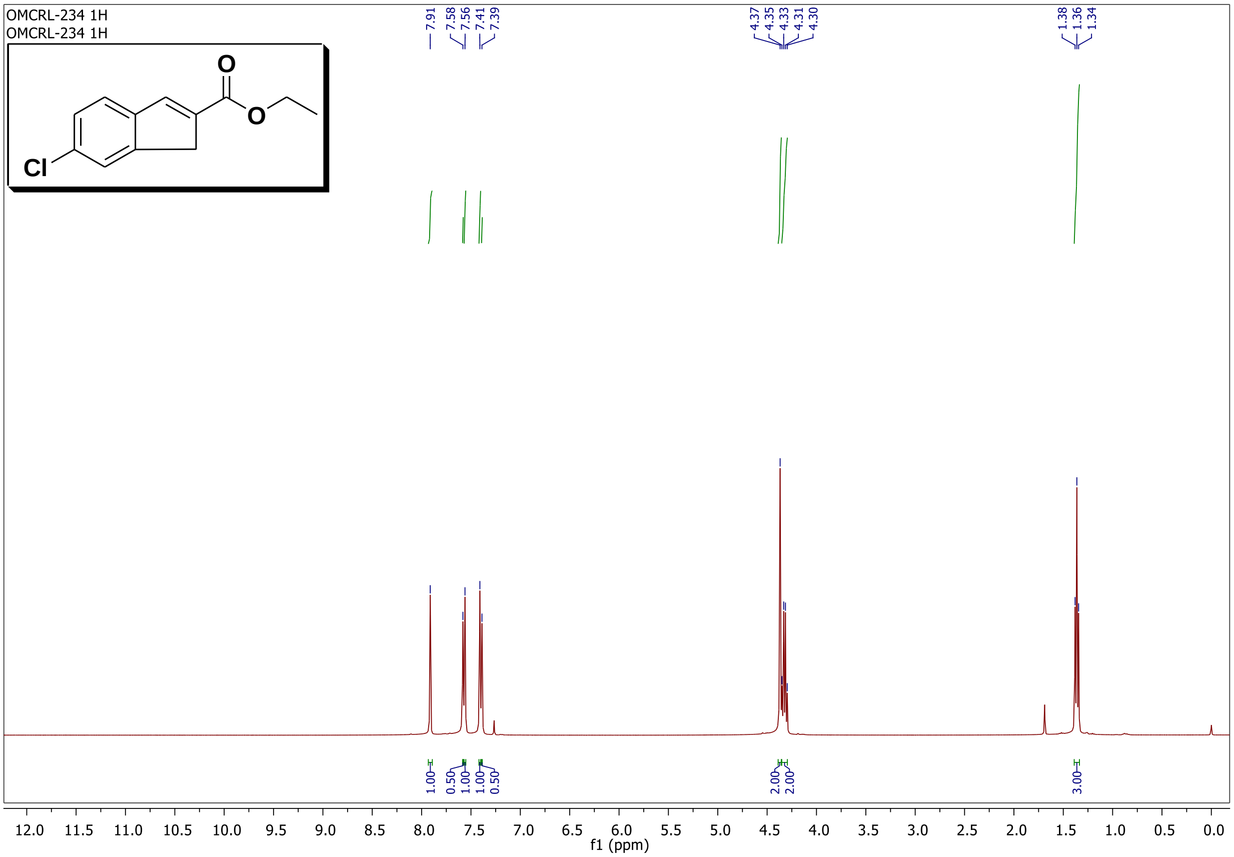

Fig. S17 ^{1}H NMR spectrum of ethyl 6-chloro-1*H*-indene-2-carboxylate (**6i**).

Carbon NMR of ethyl 6-chloro-1*H*-indene-2-carboxylate

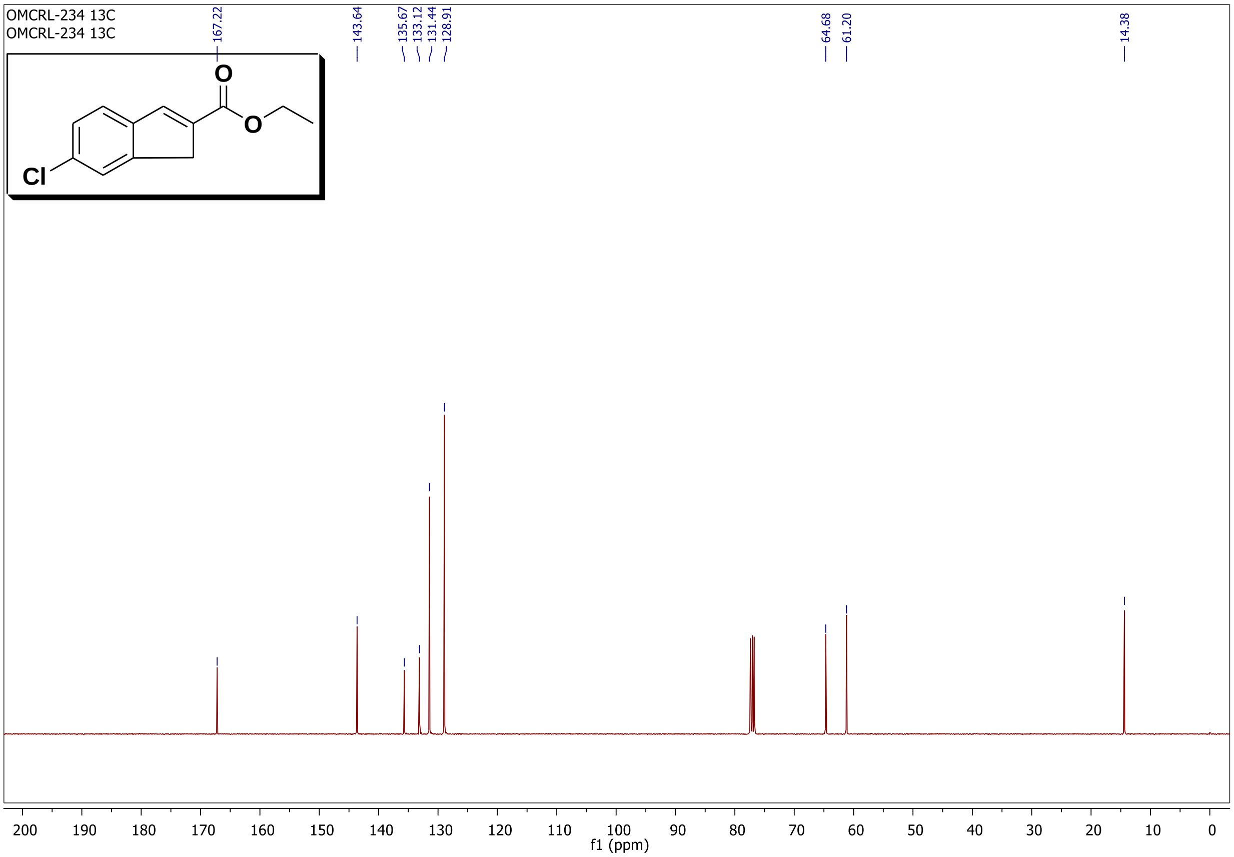

Fig. S18 ^{13}C NMR spectrum of ethyl 6-chloro-1*H*-indene-2-carboxylate (**6i**).

Proton NMR of methyl 6-fluoro-1*H*-indene-2-carboxylate

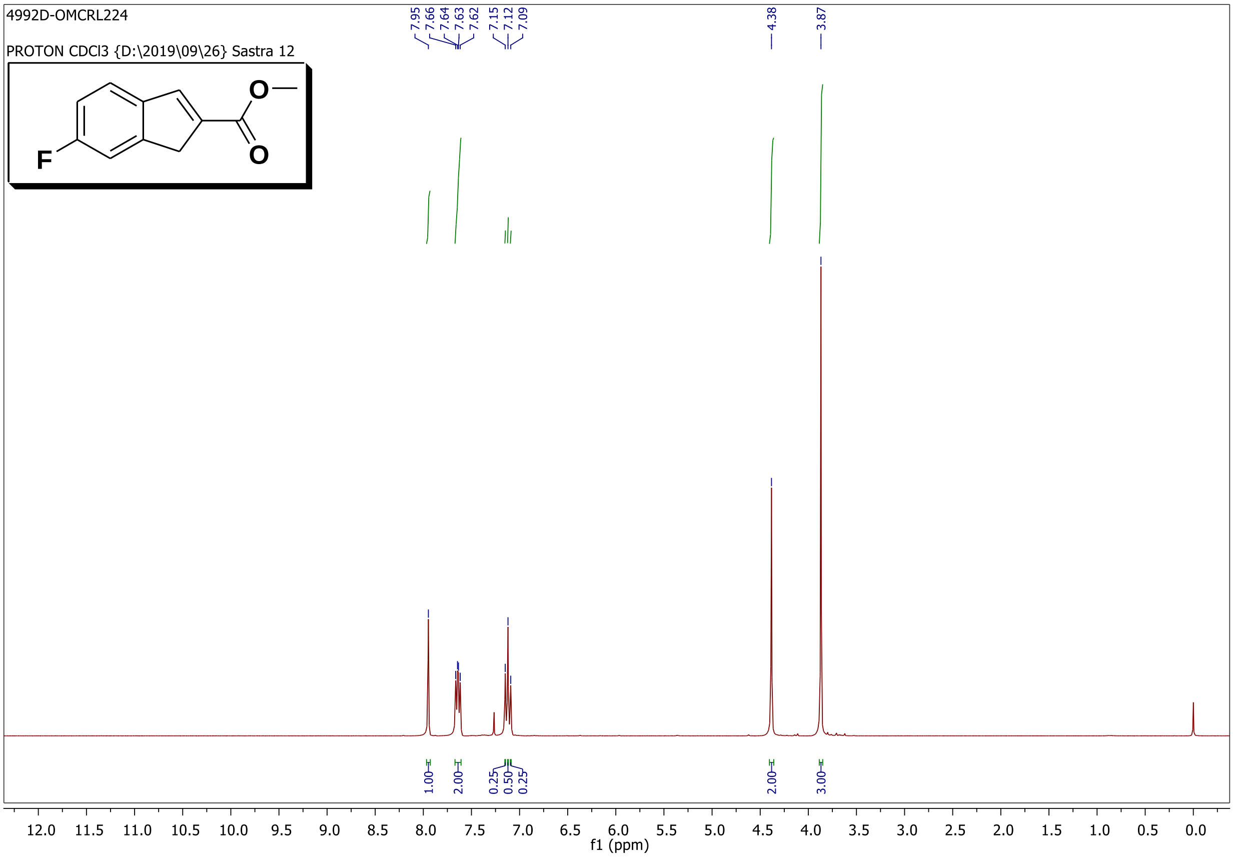

Fig. S19 ^{1}H NMR spectrum of methyl 6-fluoro-1*H*-indene-2-carboxylate (**6j**).

Carbon NMR of methyl 6-fluoro-1*H*-indene-2-carboxylate

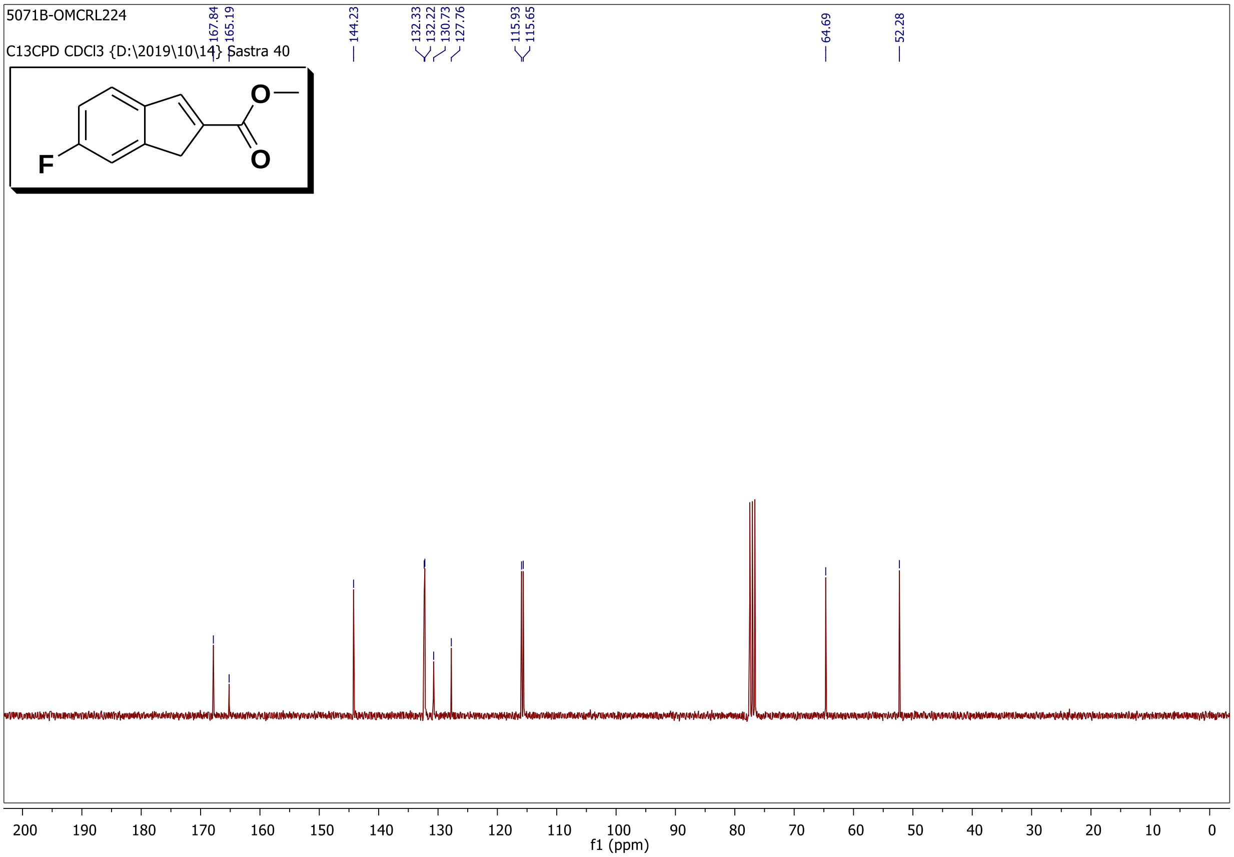

Fig. S20 ^{13}C NMR spectrum of methyl 6-fluoro-1*H*-indene-2-carboxylate (**6j**).

Proton NMR of 6-fluoro-1*H*-indene-2-carbonitrile

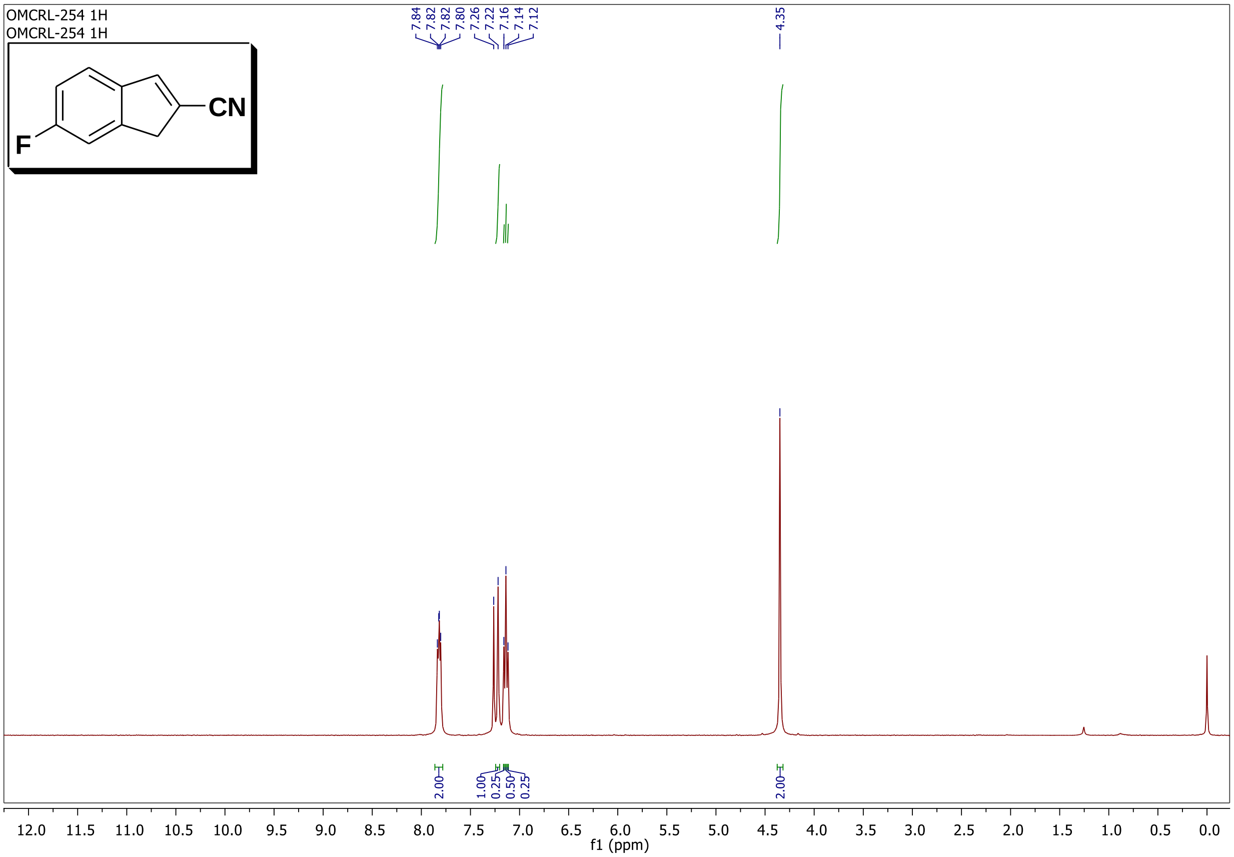

Fig. S21 ^{1}H NMR spectrum of 6-fluoro-1*H*-indene-2-carbonitrile (**6k**).

Carbon NMR of 6-fluoro-1*H*-indene-2-carbonitrile

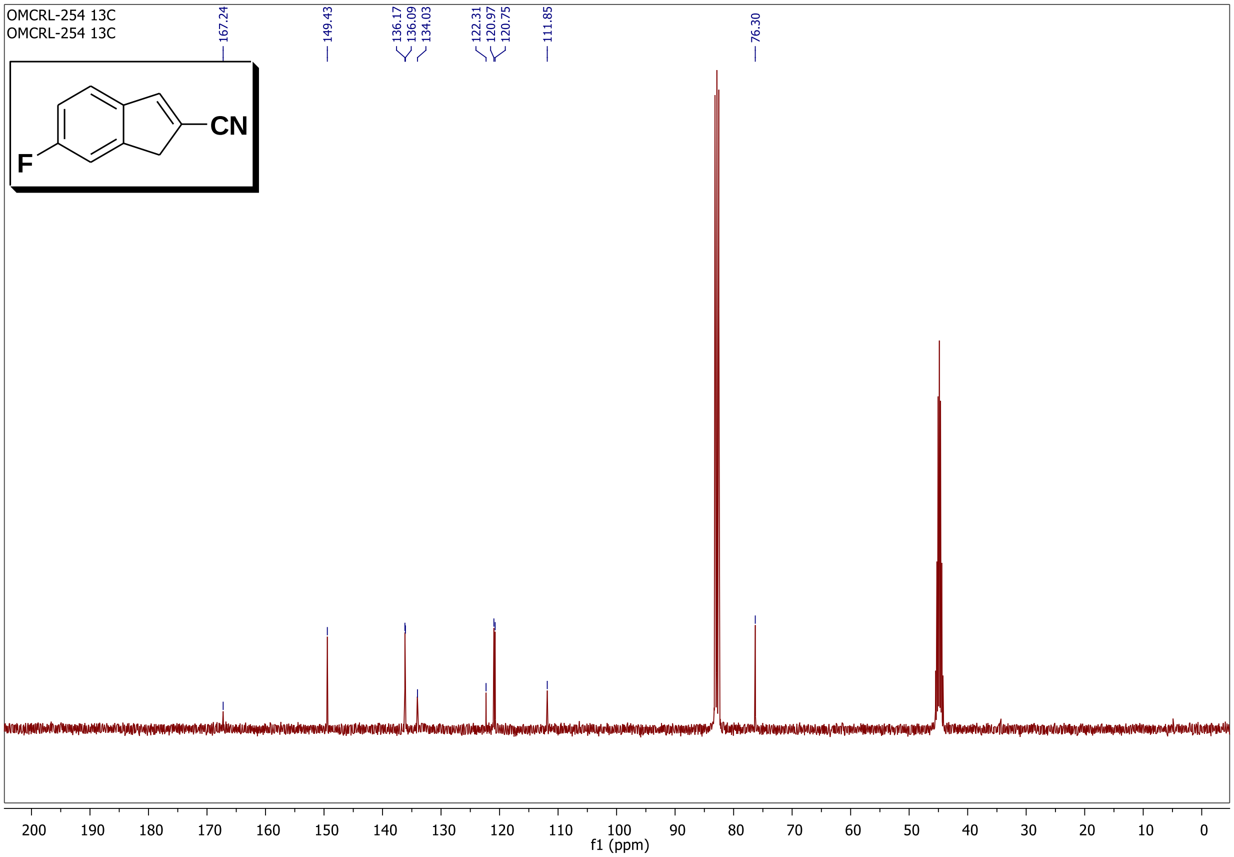

Fig. S22 ^{13}C NMR spectrum of 6-fluoro-1*H*-indene-2-carbonitrile (**6k**).

Proton NMR of ethyl 6-fluoro-1*H*-indene-2-carboxylate

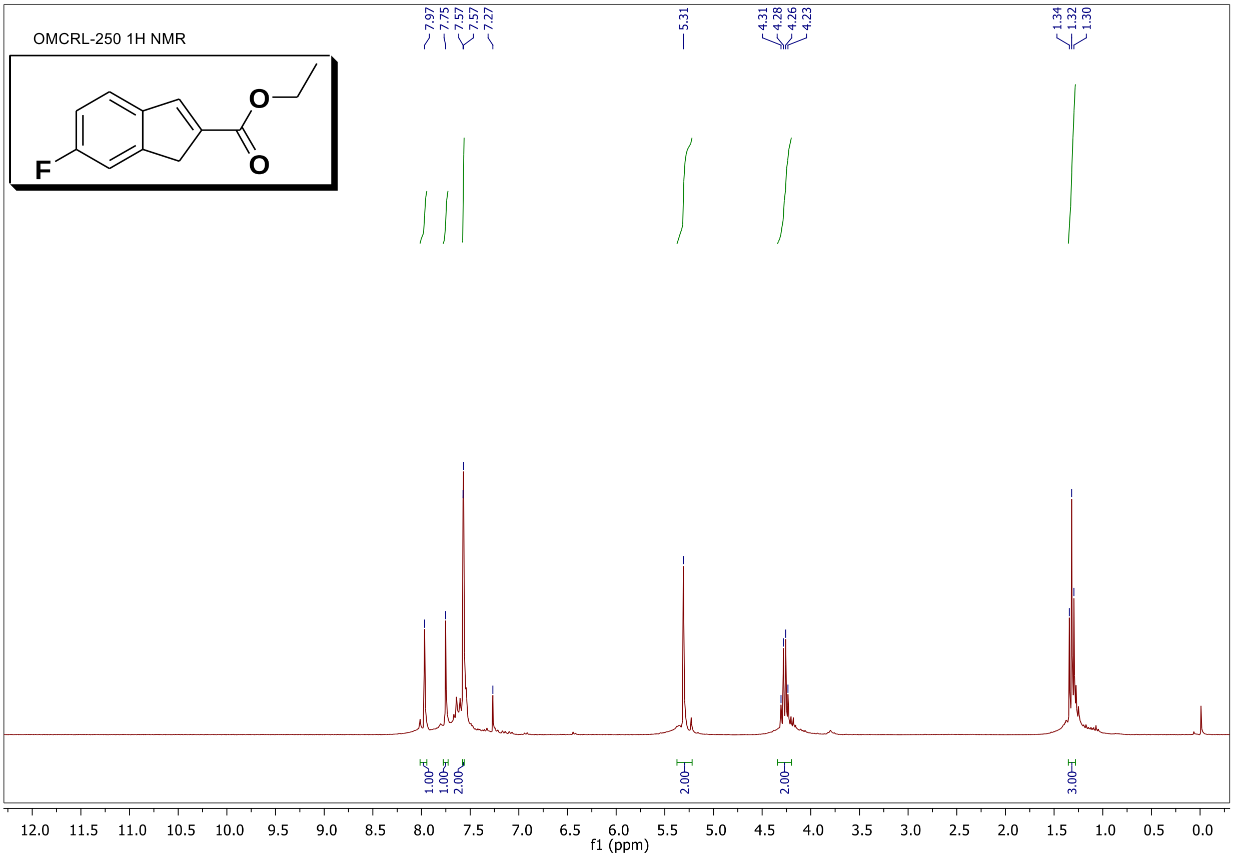

Fig. S23 ^{1}H NMR spectrum of ethyl 6-fluoro-1*H*-indene-2-carboxylate (**6l**).

Carbon NMR of ethyl 6-fluoro-1*H*-indene-2-carboxylate

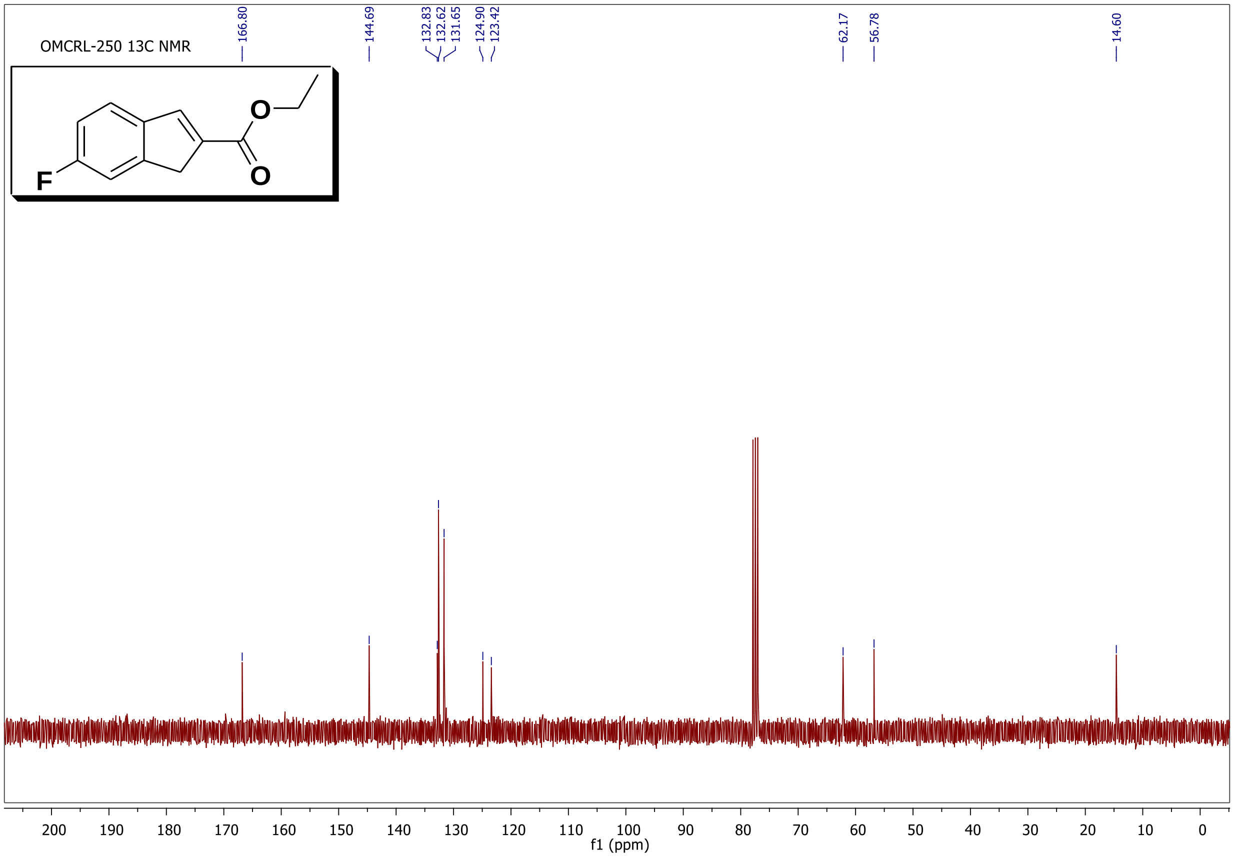

Fig. S24 ^{13}C NMR spectrum of ethyl 6-fluoro-1*H*-indene-2-carboxylate (**6l**).

Proton NMR spectrum of 1-benzylidene-3-oxopyrazolidin-1-ium-2-ide

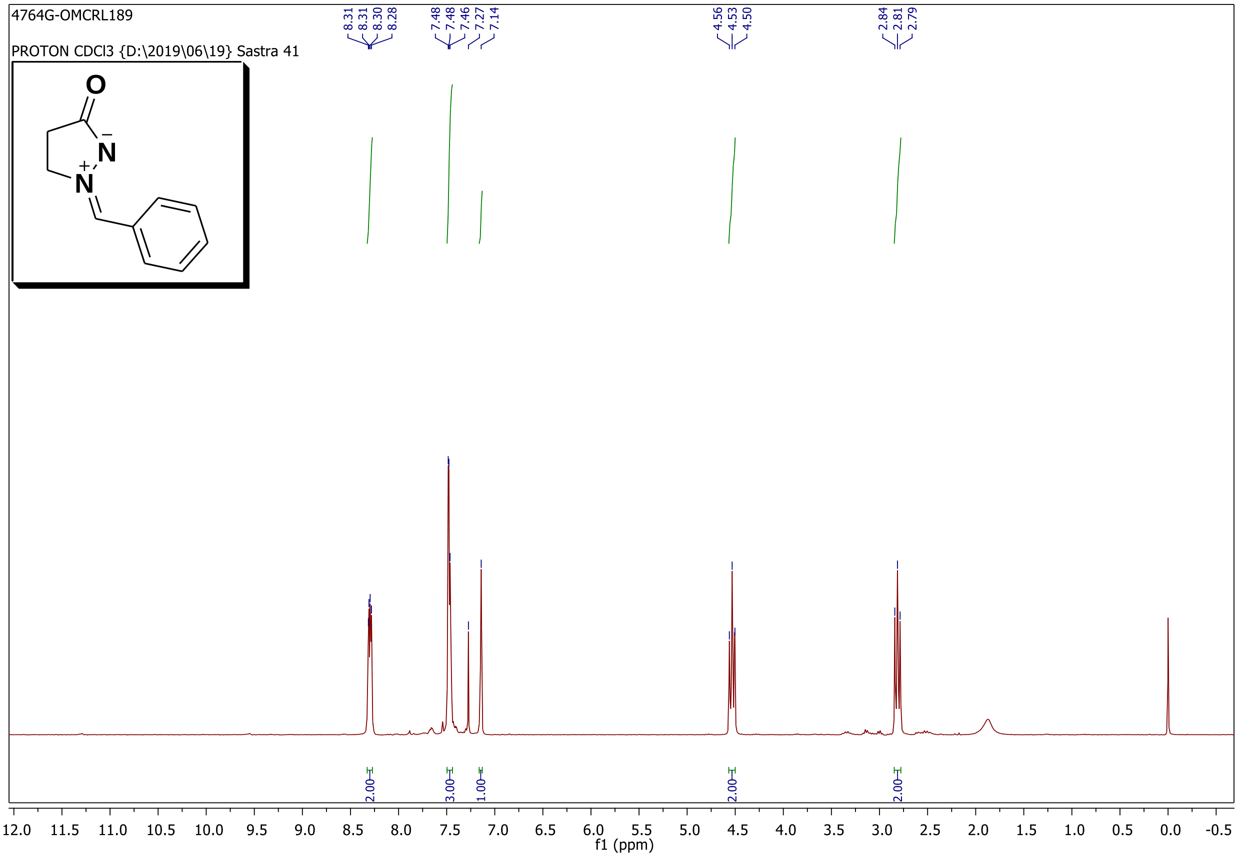

Fig. S25 ^{1}H NMR spectrum of 1-benzylidene-3-oxopyrazolidin-1-ium-2-ide (**7a**).

<u>**Carbon NMR spectrum of 1-benzylidene-3-oxopyrazolidin-1-ium-2-ide**</u>

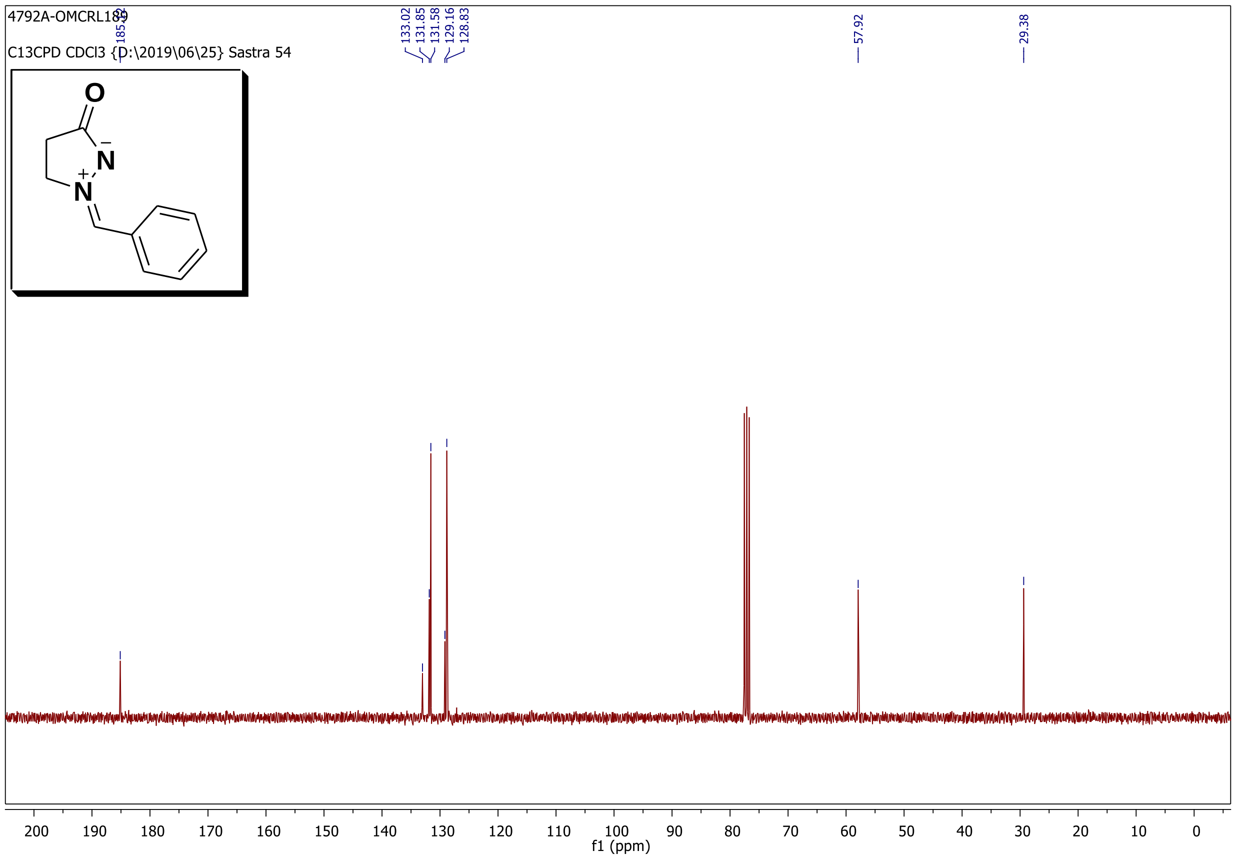

Fig. S26 ^{13}C NMR spectrum of 1-benzylidene-3-oxopyrazolidin-1-ium-2-ide (**7a**).

Proton NMR spectrum of compound 8a

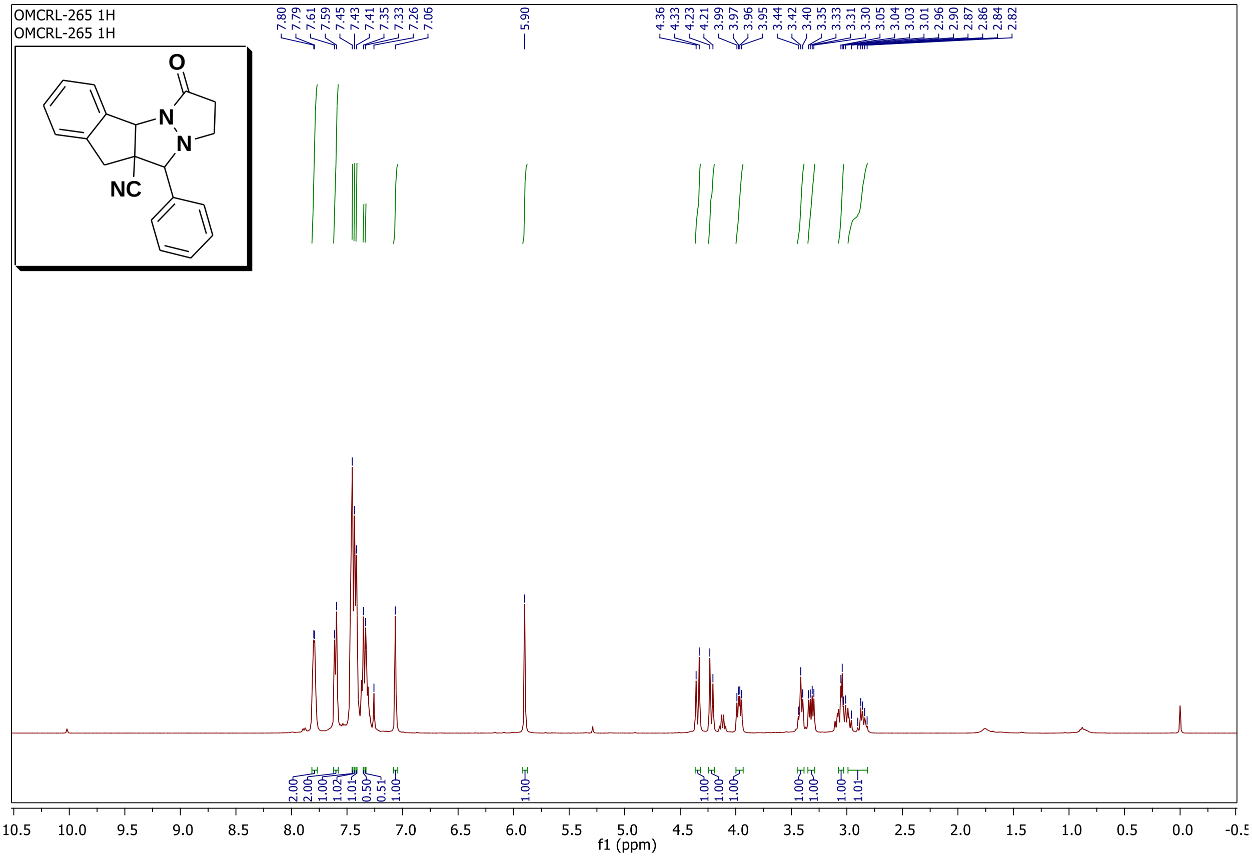

Fig. S27 ^{1}H NMR spectrum of compound **8a**.

Carbon NMR spectrum of compound 8a

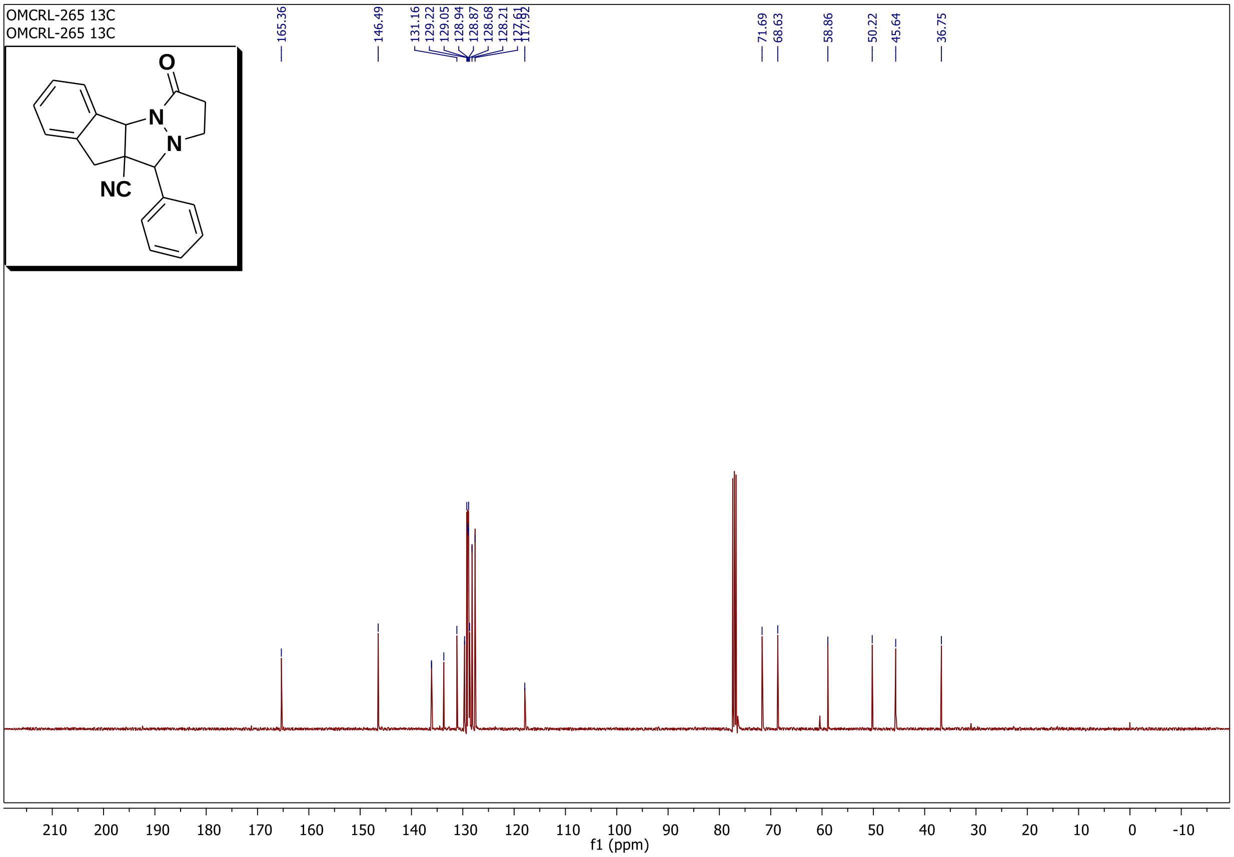

Fig. S28 ^{13}C NMR spectrum of compound **8a**.

Proton NMR spectrum of compound 8b

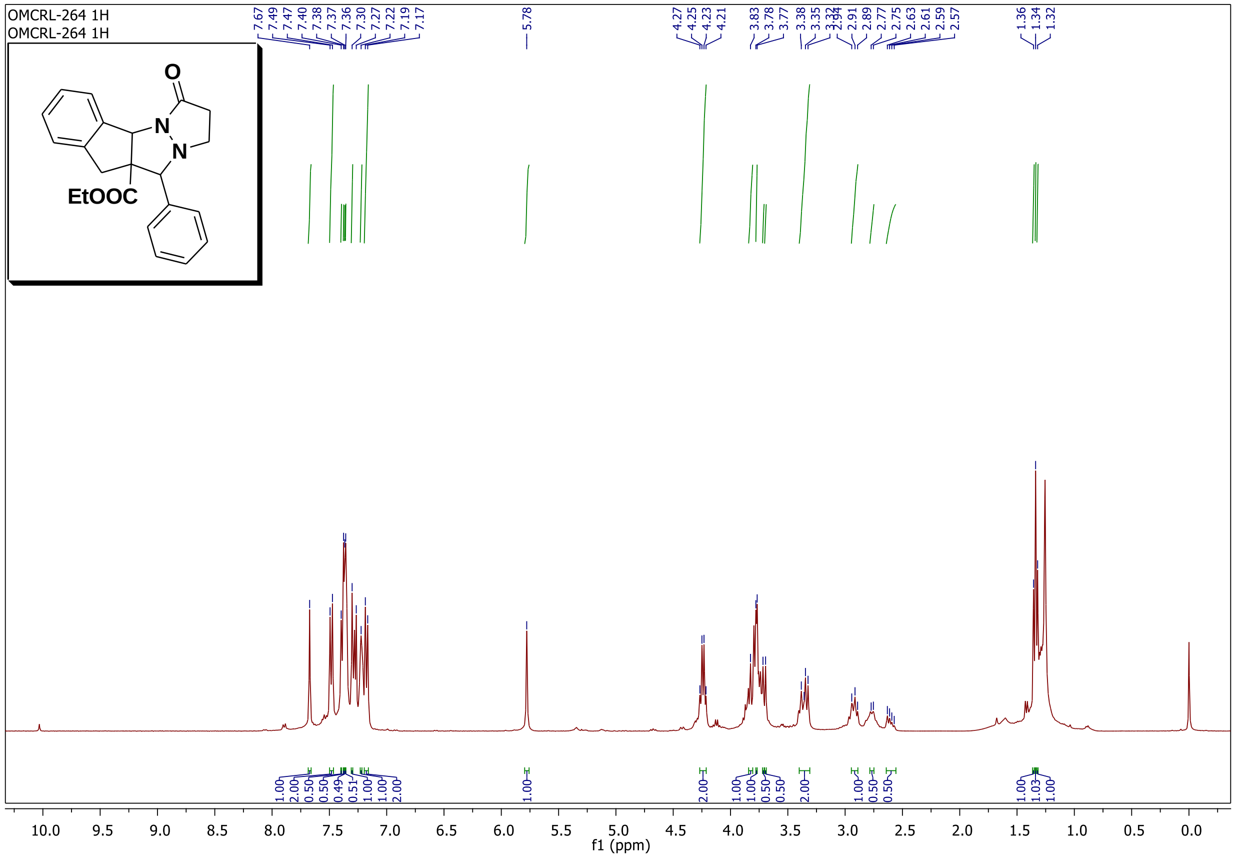

Fig. S29 ^{1}H NMR spectrum of compound **8b**.

Carbon NMR spectrum of compound 8b

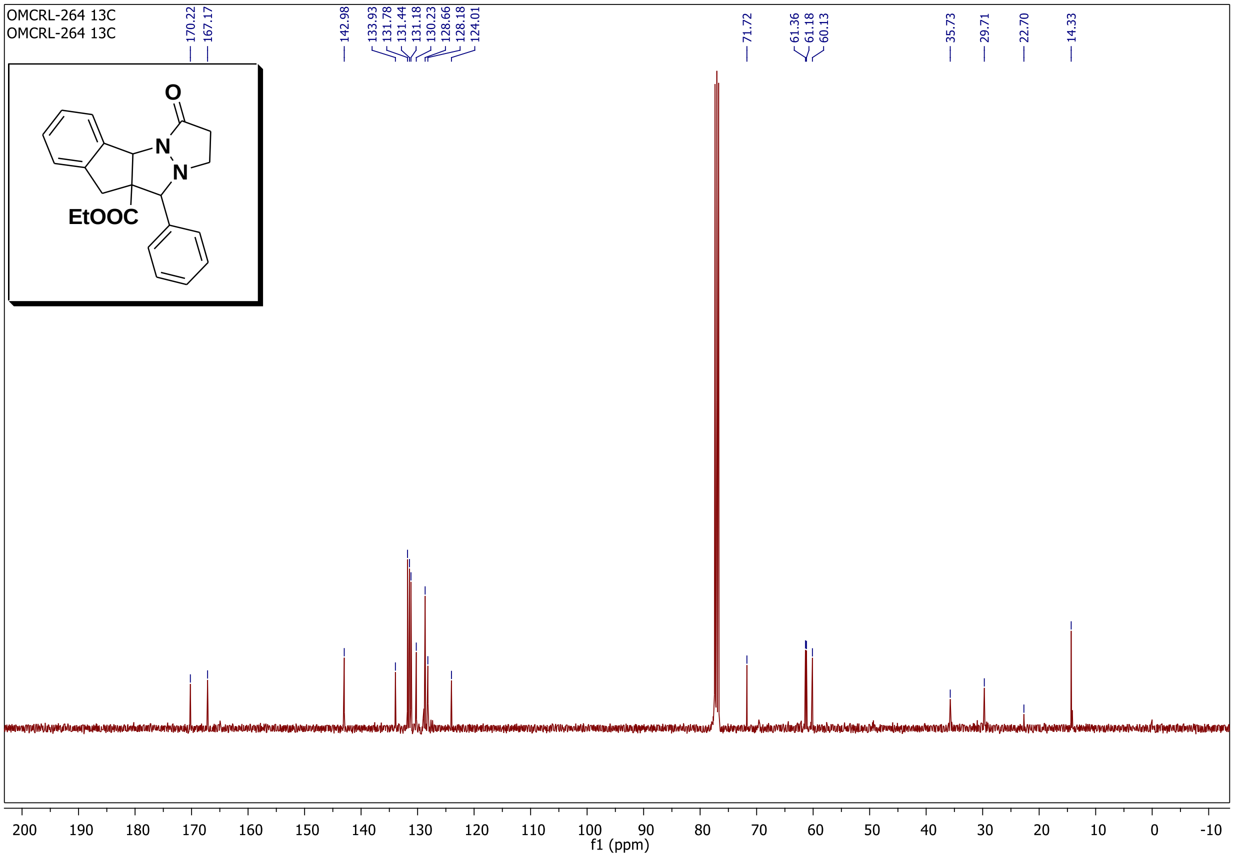

Fig. S30 ^{13}C NMR spectrum of compound **8b**.

<u>Methyl 1*H*-indene-2-carboxylate (6a)</u> [1]

Yield: 160 mg (81%); white solid; mp 85-87 °C.

IR (cm^{-1}): 3062, 2953, 2884, 1947, 1735, 1640, 1548, 1470, 1442, 1215, 734.

^1H NMR ($CDCl_3$, 300 MHz): δ_H = 7.99 (s, 1H, Alkene-CH), 7.64-7.41 (m, 4H, Aro-H), 4.41 (s, 2H, -CH_2), 3.87 (s, 3H, $COOCH_3$). ^{13}C NMR ($CDCl_3$, 300 MHz): δ_C = 168.03, 145.31, 134.67, 130.11, 129.53, 128.62, 128.25, 64.78, 52.24.

HRMS: m/z Calculated for $C_{11}H_{10}O_2$ [M+H$^+$]: 174.0681; found: 174.0706

<u>1*H*-Indene-2-carbonitrile (6b)</u> [2]

Yield: 105 mg (58%); white solid; mp 45-47 °C.

IR (cm^{-1}): 3086, 2965, 2849, 2158, 1938, 1627, 1462, 1440, 730.

^1H NMR ($CDCl_3$, 300 MHz): δ_H = 7.82-7.43 (m, 4H, Aro-H), 7.42 (s, 1H, Alkene-CH), 4.35 (s, 2H, -CH_2). ^{13}C NMR ($CDCl_3$, 300 MHz): δ_C = 145.86, 132.80, 131.00, 129.19, 129.02, 117.66, 107.18, 71.69.

HRMS: m/z Calculated for $C_{10}H_7N$ [M+H$^+$]: 141.0578; found: 141.0603

<u>Ethyl 1*H*-indene-2-carboxylate (6c)</u> [3]

Yield: 165 mg (67%); Colourless oil.

IR (cm^{-1}): 3034, 2945, 2864, 1938, 1740, 1638, 1534, 1469, 1452, 1228, 732.

^1H NMR ($CDCl_3$, 300 MHz): δ_H = 7.98 (s, 1H, Alkene-CH), 7.65-7.40 (m, 4H, Aro-H), 4.42 (s, 2H, -CH_2), 4.36-4.29 (q, J = 6Hz, 2H, $COOCH_2CH_3$), 1.38-1.34 (t, J = 6Hz, 3H, $COOCH_2CH_3$). ^{13}C NMR ($CDCl_3$, 300 MHz): δ_C = 167.57, 144.93, 134.77, 130.11, 129.44, 128.63, 128.60, 64.79, 61.06, 14.39.

HRMS: m/z Calculated for $C_{12}H_{12}O_2$ [M+H$^+$]: 188.0837; found: 188.0866

<u>Methyl 6-bromo-1*H*-indene-2-carboxylate (6d)</u>

Yield: 187 mg (74%); white solid; mp 128-130 °C.

IR (cm^{-1}): 3088, 2969, 2842, 1928, 1730, 1648, 1532, 1460, 1427, 1210, 786.

^1H NMR ($CDCl_3$, 400 MHz): δ_H = 7.90 (s, 1H, Alkene-CH), 7.58-7.48 (m, 3H, Aro-H), 4.34 (s, 2H, -CH_2), 3.87 (s, 3H, $COOCH_3$). ^{13}C NMR ($CDCl_3$, 400 MHz): δ_C = 168.02, 144.43, 133.83, 132.29, 132.00, 129.01, 124.54, 65.05, 52.74.

HRMS: m/z Calculated for $C_{11}H_9BrO_2$ [M+H$^+$]: 251.9786; found: 251.9812

<u>6-Bromo-1*H*-indene-2-carbonitrile (6e)</u>

Yield: 132 mg (60%); white solid; mp 116-118 °C.

IR (cm^{-1}): 3100, 2912, 2825, 2182, 1920, 1648, 1455, 1431, 791.

^1H NMR ($CDCl_3$, 300 MHz): δ_H = 7.69-7.57 (m, 3H, Aro-H), 7.20 (s, 1H, Alkene-CH), 4.34 (s, 2H, -CH_2). ^{13}C NMR ($CDCl_3$, 300 MHz): δ_C = 144.33, 132.30, 131.55, 130.53, 125.45, 117.26, 107.82, 71.64.

HRMS: m/z Calculated for $C_{10}H_6BrN$ [M+H$^+$]: 218.9684; found: 218.9711

Ethyl 6-bromo-1*H*-indene-2-carboxylate (6f)

Yield: 181 mg (68%); white solid; mp 98-100 °C.

IR (cm^{-1}): 3092, 2945, 2821, 1918, 1720, 1618, 1514, 1472, 1419, 1216, 776.

^{1}H NMR ($CDCl_3$, 400 MHz): δ_H = 7.89 (s, 1H, Alkene-CH), 7.57-7.48 (m, 3H, Aro-H), 4.36 (s, 2H, -CH_2), 4.33-4.27 (q, *J* = 8Hz, 2H, $COOCH_2CH_3$), 1.38-1.34 (t, *J* = 8Hz, 3H, $COOCH_2CH_3$). ^{13}C NMR ($CDCl_3$, 400 MHz): δ_C = 167.19, 143.67, 133.56, 131.88, 131.64, 129.02, 124.07, 64.68, 61.22, 14.39.

HRMS: m/z Calculated for $C_{12}H_{11}BrO_2$ [M+H$^+$]: 265.9942; found: 265.9970

Methyl 6-chloro-1*H*-indene-2-carboxylate (6g) [2]

Yield: 167 mg (80%); white solid; mp 108-110 °C.

IR (cm^{-1}): 3082, 2974, 2848, 1944, 1738, 1637, 1540, 1478, 1430, 1216, 802.

^{1}H NMR ($CDCl_3$, 300 MHz): δ_H = 7.92 (s, 1H, Alkene-CH), 7.58-7.39 (m, 3H, Aro-H), 4.35 (s, 2H, -CH_2), 3.87 (s, 3H, $COOCH_3$). ^{13}C NMR ($CDCl_3$, 300 MHz): δ_C = 168.04, 144.37, 136.13, 133.39, 131.80, 129.31, 128.88, 65.05, 52.72.

HRMS: m/z Calculated for $C_{11}H_9ClO_2$ [M+H$^+$]: 208.0291; found: 208.0319

6-Chloro-1*H*-indene-2-carbonitrile (6h)

Yield: 114 mg (65%); white solid; mp 78-80 °C.

IR (cm^{-1}): 3095, 2952, 2875, 2193, 1916, 1637, 1442, 1428, 813.

^{1}H NMR ($CDCl_3$, 300 MHz): δ_H = 7.76-7.41 (m, 3H, Aro-H), 7.21 (s, 1H, Alkene-CH), 4.35 (s, 2H, -CH_2). ^{13}C NMR ($CDCl_3$, 300 MHz): δ_C = 144.27, 137.02, 131.14, 130.39, 129.32, 117.29, 107.67, 71.63.

HRMS: m/z Calculated for $C_{10}H_6ClN$ [M+H$^+$]: 175.0189; found: 175.0218

Ethyl 6-chloro-1*H*-indene-2-carboxylate (6i) [4]

Yield: 160 mg (72%); white solid; mp 80-82 °C.

IR (cm^{-1}): 3062, 2954, 2836, 1932, 1735, 1630, 1528, 1452 , 1420, 1212, 806.

^{1}H NMR ($CDCl_3$, 400 MHz): δ_H = 7.91 (s, 1H, Alkene-CH), 7.58-7.39 (m, 3H, Aro-H), 4.37 (s, 2H, -CH_2), 4.35-4.30 (q, *J* = 8Hz, 2H, $COOCH_2CH_3$), 1.38-1.34 (t, *J* = 8Hz, 3H, $COOCH_2CH_3$). ^{13}C NMR ($CDCl_3$, 400 MHz): δ_C = 167.22, 143.64, 135.67, 133.12, 131.44, 128.91, 64.68, 61.20, 14.38.

HRMS: m/z Calculated for $C_{12}H_{11}ClO_2$ [M+H$^+$]: 222.0448; found: 222.0476

Methyl 6-fluoro-1*H*-indene-2-carboxylate (6j) [2]

Yield: 163 mg (85%); white solid; mp 113-115 °C.

IR (cm^{-1}): 3094, 2983, 2892, 1965, 1740, 1628, 1558, 1462, 1449, 1242, 812.

^1H NMR ($CDCl_3$, 300 MHz): δ_H = 7.95 (s, 1H, Alkene-CH), 7.66-7.09 (m, 3H, Aro-H), 4.38 (s, 2H, -CH_2), 3.87 (s, 3H, $COOCH_3$). ^{13}C NMR ($CDCl_3$, 300 MHz): δ_C = 167.75, 165.19, 144.23, 132.33, 132.22, 130.73, 127.76, 115.93, 115.65, 64.69, 52.28.

HRMS: m/z Calculated for $C_{11}H_9FO_2$ [M+H$^+$]: 192.0587; found: 192.0617.

6-Fluoro-1*H*-indene-2-carbonitrile (6k)

Yield: 110 mg (69%); white solid; mp 105-107 °C.

IR (cm^{-1}): 3098, 2968, 2884, 2199, 1928, 1646, 1456, 1436, 820.

^1H NMR ($CDCl_3$, 400 MHz): δ_H = 7.82 (s, 1H, Alkene-CH), 7.22-7.12 (m, 3H, Aro-H), 4.35 (s, 2H, -CH_2). ^{13}C NMR (DMSO-D_6, 300 MHz): δ_C = 167.24, 149.43, 136.17, 136.09, 134.06, 122.31, 120.97, 120.75, 111.85, 76.30.

HRMS: m/z Calculated for $C_{10}H_6FN$ [M+H$^+$]: 159.0484; found: 159.0513

Ethyl 6-fluoro-1*H*-indene-2-carboxylate (6l)

Yield: 156 mg (76%); white solid; mp 110-112 °C.

IR (cm^{-1}): 3092, 2984, 2896, 1962, 1738, 1662, 1542, 1478, 1446, 1245, 816.

^1H NMR ($CDCl_3$, 400 MHz): δ_H = 7.97 (s, 1H, Alkene-CH), 7.75-7.57 (m, 3H, Aro-H), 5.31 (s, 2H, -CH_2), 4.31-4.23 (q, *J* = 8Hz, 2H, $COOCH_2CH_3$), 1.34-1.30 (t, *J* = 8Hz, 3H, $COOCH_2CH_3$). ^{13}C NMR ($CDCl_3$, 300 MHz): δ_C = 166.80, 144.69, 132.83, 132.62, 131.65, 124.90, 123.42, 62.17, 56.78, 14.60.

HRMS: m/z Calculated for $C_{12}H_{11}FO_2$ [M+H$^+$]: 206.0743; found 206.0773.

Synthesis of 1-benzylidene-3-oxopyrazolidin-1-ium-2-ide (7a) [5, 6]

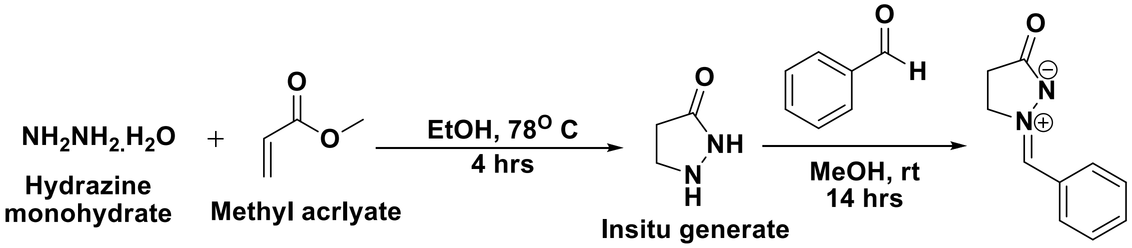

(7a)

Scheme. 1 Synthesis of 1-Benzylidene-3-oxopyrazolidin-1-ium-2-ide (7a).

1-Benzylidene-3-oxopyrazolidin-1-ium-2-ide (7a)

Yield: 132 mg (67%); Off white solid; mp 194-196 °C.

IR (cm^{-1}): 1674, 1652, 1582, 1568, 1454, 1278, 1117

^{1}H NMR ($CDCl_3$, 400 MHz): δ_H = 8.31-7.46 (m, 5H, Aro-H), 7.14 (s, 1H, N=CH), 4.56-4.50 (t, *J* = 8Hz, 2H, CO-CH_2), 2.84-2.79 (t, *J* = 8Hz, 2H, N-CH_2). ^{13}C NMR ($CDCl_3$, 400 MHz): δ_C = 185.12 (1C, C=O), 133.02- 128.83 (6C, Aro-C), 57.92 (1C, N-CH_2), 29.38 (1C, CO-CH_2).
HRMS: m/z Calculated for $C_{10}H_{10}N_2O$ [M+H^+]: 174.0793; found: 174.0818.

Cycloaddition compound 8a

Yield: 192mg (61%); yellowish oil.

IR (cm^{-1}): 2972, 2254, 1954, 1562, 1671, 1455, 1245.

^{1}H NMR ($CDCl_3$, 400 MHz): δ_H = 7.80-7.06 (m, 9H, Aro-*H*), 5.90 (s, 1H, *H*C-N-CO), 4.36-4.21 (m, 2H, Aro-CH_2), 3.99-3.95 (m, 1H, Aro-C*H*-N-N), 3.44-3.30 (m, 2H, N-CH_2-CH_2), 3.05-2.82 (m, 2H, N-CH_2-CH_2). ^{13}C NMR ($CDCl_3$, 400 MHz): δ_C = 165.36, 146.49, 136.10, 133.70, 131.16, 129.66, 129.22, 129.05, 128.94, 128.87, 128.68, 128.21, 127.61, 117.92, 71.69, 68.63, 58.86, 50.22, 45.64, 36.75.
HRMS: m/z Calculated for $C_{20}H_{17}N_3O$ [M+H^+]: 315.1372; found: 315.1397.

Cycloaddition compound 8b

Yield: 260 mg (72%); yellowish oil.

IR (cm^{-1}): 2984, 1962, 1676, 1585, 1463, 1453, 1320, 1257.

^{1}H NMR ($CDCl_3$, 400 MHz): δ_H = 7.67-7.17 (m, 9H, Aro-*H*), 5.78 (s, 1H, *H*C-N-CO), 4.27-4.21 (q, *J* = 8Hz, 2H, -COOC$H_2$$CH_3$), 3.83 (s, 1H, Aro-C*H*-N-N), 3.78-3.69 (m, 2H, Aro-CH_2), 3.38-3.32 (m, 2H, N-CH_2-CH_2), 2.94-2.57 (m, 2H, N-CH_2-CH_2), 1.36-1.32 (t, *J* = 8Hz, 2H, -COOCH_2CH_3). ^{13}C NMR ($CDCl_3$, 300 MHz): δ_C = 170.22, 167.17, 142.98, 133.93, 131.78, 131.44, 131.18, 130.23, 128.66, 128.18, 124.01, 71.72, 61.36, 61.18, 60.13, 35.73, 29.71, 22.70, 14.33.

HRMS: m/z Calculated for $C_{22}H_{22}N_2O_3$ [M+H^+]: 362.1630; found: 362.1657.

References

1. Chang, K. -J.; Rayabarapu, D. K.; Cheng, C. –H. *J. Org. Chem.*, **2004**, *69*, 4781-4787. DOI: 10.1021/jo049506g

2. Das, B. G.; Chirila, A,; Tromp, M.; Reek, J. N. H.; de Bruin, B. *J. Am. Chem. Soc.*, **2016**, *138*, 8968-8975. DOI: 10.1021/jacs.6b05434

3. Seomoon, D.; Lee, K.; Kim, H.; Lee, P. H. *Chem. Eur. J.*, **2007**, *13*, 5197-5206. DOI: 10.1002/chem.200601338

4. Sruthi, P. R.; Saranya, T. V.; Anas, S. *ChemistrySelect* **2020**, *5*, 1648-1654.5. DOI: 10.1002/slct.201903515

5. Shintani, R.; Fu, G. C. *J. Am. Chem. Soc.*, **2003**, *125*, 10778-10779. DOI: 10.1021/ja036922u

6. Keller, M.; Sido, A. S. S.; Pale, P.; Sommer, J. *Chem. Eur. J.*, **2009**, *15*, 2810-2817. DOI: 10.1002/chem.200802191
